# Perilymphatic ATP plays a critical role in modulating cochlear function to protect from loud sound induced hearing loss

**DOI:** 10.1016/j.ebiom.2026.106377

**Published:** 2026-07-14

**Authors:** Sonal Prasad, Urban Karlsson, Marja Pitkänen, Anders Fridberger

**Affiliations:** Department of Biomedical and Clinical Sciences, Linköping University, Linköping, SE-581 83, Sweden

**Keywords:** Loud sound, Protection, Hearing loss, ATP, Sensorineural, Calcium

## Abstract

**Background:**

Extracellular adenosine triphosphate (ATP) signalling via purinergic receptors plays a key role in cochlear adaptation to loud sound. Traditionally, ATP activation of purinergic receptor P2X receptors has been proposed to induce a cation shunt, reducing the endolymphatic potential and the driving force for sound transduction. However, direct evidence for this protective mechanism remains limited. Here, we provide direct experimental evidence identifying a distinct, compartment-specific ATP signalling pathway that modulates cochlear function.

**Methods:**

In mature Dunkin–Hartley guinea pigs of either sex, we combined time-resolved confocal microscopy, electrophysiology, live-cell imaging, and fluorescence spectroscopy to characterise how compartmentalised extracellular ATP regulates cochlear function during moderate loud sound exposure.

**Findings:**

ATP delivered to the perilymphatic space where P2X2 receptors localised in Reissner's membrane epithelial cells, supporting cells, and hair cells significantly reduced sound-evoked electrical potentials from 486 μV to 315 μV, outer hair cell stereocilia motion from 135 nm to 99 nm and Hensen's cell motion from 128 nm to 101 nm at 80 dB SPL. These effects were reversible upon ATP removal and accompanied by decreased intracellular calcium. In contrast, ATP applied to the endolymph produced no comparable changes. These findings demonstrate that extracellular ATP in the perilymph protects the cochlea from high-intensity sound through a mechanism distinct from the classical cation shunt model.

**Interpretation:**

These findings reveal a previously unrecognised perilymph-driven ATP signalling pathway that extend beyond the traditional P2X-mediated cation shunt, demonstrating that extracellular ATP plays a critical role in protecting the cochlea mainly from loud sound-induced hearing loss.

**Funding:**

Swedish Research Council 2017-06092 and 2022-00548.

**Research in context**

**Evidence before this study**

Extracellular ATP is known to modulate cochlear responses to acoustic stress, primarily through ATP release and activation of P2X2 receptors reported during loud sound exposure. Genetic studies, particularly in P2X2-knockout mice, show increased vulnerability to noise-induced hearing loss, supporting a protective role for ATP signalling. The prevailing model and dominant hypothesis suggest that ATP induces a cation shunt that lowers the endolymphatic potential and reduces the driving force for mechanoelectrical transduction, thereby offering protection against acoustic overstimulation. However, direct experimental evidence supporting this mechanism has remained insubstantial, and the spatial specificity of ATP actions within the cochlea to provide protection against loud sound have not been systematically examined and hence not fully resolved.

**Added value of this study**

This study provides the direct demonstration that extracellular ATP in the perilymph, rather than the endolymph, modulates cochlear mechanics and electrophysiology during loud sound exposure. We used a physiologically based animal model to investigate what happens to cochlea during high intensity sound and how it is protected. Using time-resolved confocal microscopy, electrophysiology, live-cell imaging, and fluorescence spectroscopy in mature guinea pigs, we discovered that perilymphatic ATP reduces sound-evoked electrical potentials, outer hair cell stereocilia motion largely at 80–85 dB SPL. We found that these effects are reversible and accompanied by decreased intracellular calcium levels. To confirm our findings, we applied ATP to the endolymph which did not produce similar changes. These findings identify a previously unrecognised, compartment-specific ATP signalling pathway that operates independently of the classical cation shunt model.

**Implications of all the available evidence**

The evidence demonstrates extracellular ATP in the perilymph as a critical modulator of cochlear resilience to high-intensity sound, this study reframes current understanding of purinergic signalling in auditory physiology. The available findings challenge the current role of ATP in offering protection by inducing cation shunt and highlight the importance of compartmentalised ATP dynamics in cochlear protection against overstimulation. The newly established understanding on perilymph-driven ATP pathway offers a mechanistic foundation for developing better diagnostic tools and treatments that target not just sensory cells, but also the supporting structures involved in sound transduction to prevent or mitigate loud-sound induced hearing loss.

## Introduction

Extracellular adenosine triphosphate (ATP) acts as a key signalling molecule in the cochlea, where it modulates auditory sensitivity and contributes to protective mechanisms against acoustic overstimulation.^1^ ATP exerts its effects primarily through purinergic receptors, including the ionotropic P2X family. Among these, the P2X2 receptor subtype is prominently expressed in cochlear sensory and supporting cells, particularly in regions exposed to the perilymph and endolymph.^2^ These receptors form ATP-gated cation channels that are activated during periods of intense auditory stimulation, suggesting a role in cochlear adaptation and homoeostasis.^1^

Mice lacking these ionotropic ATP receptors show increased vulnerability to acoustic trauma, highlighting their protective function.^3^^,^^4^ The importance of P2X2 receptors is further supported by human genetic studies, which link mutations in these receptors to progressive sensorineural hearing loss, likely due to altered ATP sensitivity.^1^

Previous studies demonstrated that ATP is released to the extracellular space through gap junctional hemichannels^5^^,^^6^ and Panx1 channels.^7^ It then activates P2X2 receptors, particularly in Reissner's membrane, creating a cation shunt that reduces the positive electrical potentials.^8^^,^^9^ This diminishes the electrochemical driving force for ion entry into hair cells, thereby lowering receptor currents and cellular stress during loud sound.

Activation of P2X receptors also increases intracellular calcium in hair and supporting cells, suggesting a broader modulatory role in cochlear function such as neurotransmission, cytoskeletal dynamics, and gene expression.^10^^,^^11^ Increase of intracellular calcium concentration upon activation of P2X receptors induces hair cell depolarisation and release of glutamate vesicles. This may contribute to regulation of sound transduction similar to what shown at a tissue level when ATP released in endolymphatic compartment. However, much of the supporting evidence comes from isolated cell studies, which may not accurately reflect the behaviour of intact cochlear preparations due to loss of epithelial polarity.^12^^,^^13^

Notably, Kujawa et al., (1994)^14^ demonstrated that ATP and related agonists, when applied to the cochlear perilymph, suppressed cochlear electrical potentials and distortion product otoacoustic emissions (DPOAEs) even at low sound levels. ATP and P2X2 receptors are known to modulate cochlear responses and contribute to hearing protection during loud sound exposure. However, the mechanisms underlying ATP-mediated effects in the cochlea particularly whether they originate primarily from the endolymphatic or perilymphatic compartments and how they depend on sound intensity are not yet fully understood.

We hypothesised that ATP levels in the perilymph rise transiently during varied loud sound exposure and contribute to cochlear protection. To test this, we applied ATP selectively to the endolymphatic and perilymphatic compartments during moderate and loud sound exposure.

The aim of the study was to define how compartmentalised extracellular ATP modulates cochlear sensitivity during moderate loud sound exposure and to identify the specific signalling pathway underlying this protective mechanism. Our findings showed that ATP in the perilymph, but not in the endolymph, reduced sound-evoked responses through ATP P2X2 receptor activation, and that this effect emerged largely at high sound levels.

## Methods

### Ethics statement

The Regional Ethics Board in Linköping approved all animal experiments (DNR 5111-2019). Animal care was under the supervision of the Unit for Laboratory Animal Science at Linköping University, ensuring compliance with Swedish regulations on the care and use of animals. Animals were housed in facilities approved by the Swedish Ministry of Agriculture. All animals had free access to food and water, cages featured an enriched environment. Animals that showed signs of distress according to a standardised assessment scale were euthanised. The reporting here conforms to the ARRIVE guidelines (https://arriveguidelines.org).

### Animal and experimental model details

Young mature Dunkin-Hartley guinea pigs of both sexes (250–350 g; 5–6 weeks old) were used for all experiments (guinea pigs have low-frequency hearing that is similar to the human one). Prior to decapitation, all animals were tested for Preyer reflex and then anaesthetised with 18–24 mg of sodium pentobarbital intraperitoneally, according to their body weight. The left temporal bone was excised and attached to a custom-built holder. The holder allowed immersion of the cochlea and the middle ear in oxygenated (95% O_2_, 5% CO_2_) DMEM tissue culture medium. The bone of the bulla was gently removed to expose the middle ear and the cochlea, including the round window niche. Thereafter, a small triangular or trapezoidal opening was made in scala vestibuli in the apex and a 0.6 mm diameter hole was drilled in scala tympani in the base of the cochlea. These openings allowed continuous perfusion of oxygenated tissue culture medium, dyes or pharmacological substances through an external syringe tube connected to the basal hole with a plastic microtube. Sound stimulation was generated with a calibrated loudspeaker and delivered to the middle ear through a plastic tube. Because of the immersion of the middle ear and the opening at the apex, the effective sound stimulus was reduced by ∼20 dB.^15^ The sound pressure level values given throughout the text are corrected for this attenuation. The whole preparation was maintained at room temperature (22–24 °C). The apical opening allowed confocal imaging of the hearing organ and permitted insertion of a glass microelectrode filled with artificial endolymph-like solution (1.3 mM NaCl, 31 mM KHCO_3_, 23 μM CaCl_2_, 128.3 mM KCl, pH 7.4 and 300 mOsmol/kg, adjusted with sucrose) into the scala media through the Reissner's membrane. This microelectrode was used for cochlear microphonic recordings, summating potential recordings, electrical stimulation, endocochlear potential recordings, injection of dyes and delivery of pharmacological substances, as specified. This kind of *ex vivo* preparation does not fully retain the active mechanisms that exist *in vivo*, but basic aspects of sensory transduction are intact and functioning. The isolated preparation retains the passive mechanics of the hearing organ which makes the preparation useful for investigating cochlear functionality in a nearly native environment under various physiological modified situations. *Ex vivo* preparations offer direct optical access, precise mechanical stimulation, and controlled pharmacology that are difficult to achieve *in vivo*.

### Reagents

The following stock solutions were prepared and further diluted in artificial endolymph to the desired concentration. Di-3-ANEPPDHQ (30,315 Potentiometric Probes): 4 mM in pure DMSO diluted 100 times for use. Calcein-AM, cell permeant dye (C1430 ThermoFisher Scientific): 4 mM in pure DMSO diluted 100 times for use. Yo-Pro-1 Iodide (Y3603 ThermoFisher Scientific): 1 mM in pure DMSO diluted 100 times for use. Lucifer Yellow CH, Lithium Salt (L453 ThermoFisher Scientific): Dissolved in water and diluted to 0.2% for use. ATP disodium salt hydrate (J61125 ThermoFisher Scientific): 10 mM in clear DMEM medium and diluted to 1 mM for use when perfused and 5 mM when injected. TNP-ATP (T7602 ThermoFisher Scientific): 10 mM in clear DMEM medium and diluted to 100 μM for use. Note that the effective concentration in the endolymph is lower than mentioned because the agent is diluted in the scala media fluids upon injection. Previous estimates suggest a 10x dilution factor.^16^

We used multi-disciplinary approach, combining integrated time-resolved confocal microscopy and electrophysiological recordings of sound-evoked responses, live-cell confocal imaging to assess morphological changes, interferometry to quantify sound-evoked cochlear vibrations, fluorescence spectroscopy to measure calcium dynamics, and patch-clamp electrophysiology to evaluate ATP-dependent changes in cellular currents. Together, these approaches were used to determine how compartmentalised extracellular ATP regulates cochlear function.

### Electrophysiological recordings

Glass capillary microelectrodes with an outer diameter of 1.5 mm were pulled with a standard electrode puller and bevelled at 20° to a final resistance of ∼3–6 MΩ. The microelectrodes with 3 μm tip diameter were mounted in a manual micromanipulator at an angle of 30° and positioned in the apical opening using a 5x, 0.25 NA air objective lens (Zeiss FLUAR). Reissner's membrane was penetrated using a hydraulic stepping motor. Current injections were performed with a linear stimulus isolator (A395, World Precision Instruments) sending positive steady state currents of up to + 10 μA. These currents restored the normal potential around the hair bundles, leading to an increase of the currents through the MET channel, and in the force produced by the hair cells. The baseline average endocochlear potential upon penetration of Reissner's membrane was ∼+10–20 mV which is 40–50% of the value recorded at the apex in live animals (+50–60 mV).^17^ Cochlear microphonic potentials were recorded with chlorided Ag wires placed inside the glass capillary and in the culture medium contiguous with scala tympani. The signal was amplified 10x with an Ix1 amplifier (Dagan Instruments) and digitised with a 24-bit A/D board (USB-4431, National Instruments) at 10 kHz, using custom Labview software. Tuning curves were obtained by applying a series of tone bursts at 85 dB SPL and 65 dB SPL ranging from 60 to 820 Hz. The rise and fall time was 1 ms, using a Hanning window. The sampled signals were Fourier-transformed, and the peak amplitude was plotted as a function of stimulus frequency. Before applying ATP, tuning curve measurements were repeated every 5 min for 15 min to verify that the response was stable. We thereafter proceeded with the examinations described below. Data acquired in the *ex vivo* preparation have sharper tuning curve peaks because of the opening of the cochlear bone at the apical site, which reduces the response at very low stimulus frequencies.^18^

### Time-resolved rapid confocal imaging

To measure sound-evoked outer hair cell bundle motion, the hearing organ was stained with 2 μl of cell membrane dye di-3- ANEPPDHQ and 2 μl of Calcein-AM added in the perfusion. Dye di-3-ANEPPDHQ was applied via perfusion and injection to stain the membrane of hair cell, stereocilia bundle and supporting cells and Reissner's membrane during experiments. Subsequently, the sensory hair cell bundles were stained with di-3-ANEPPDHQ dissolved in the electrode solution and delivered to the hair bundles iontophoretically with a current stimulus of 5–10 μA or with a brief pressure injection for 5 s at 10 pound-per-square inch pressure pulse (psi) using Pico-spritzer. This protocol ensured minimal dye release into the scala media and produced strong labelling of stereocilia while preserving the barrier function of Reissner's membrane. During 60 s following the injection, it was ensured that the injection was successful, and the dye did not diffuse into the scala vestibuli. Preparations that had significant dye diffusion into the scala vestibuli because of ruptured Reissner's membrane or leakage next to electrode were discarded. To determine outer hair cell bundle displacement movements, images were acquired with a 40x, 0.80 NA (Nikon CFI Apo lens) water immersion objective lens with appropriate optical emission filter. The argon laser line at 488 nm and matching beamsplitter (MBS 488/561) was used. To avoid bleaching, the laser power was set to the minimum value consistent with an acceptable signal-to-noise ratio. The preparation was stimulated acoustically near the bundles' best frequency (180–220 Hz). The best frequency was selected from the highest peak of the tuning curve of the cochlear microphonic recordings. Image acquisition triggered both the acoustic and electrical stimulus.^19^ A series of 12 images was acquired for sound stimulus while a series of 37 images; each series requiring ∼40 s was acquired for combined sound and current stimulus, especially to visualise electrically evoked motion. For combined sound and electrical stimuli, current injection switched directly from positive to negative at 5 Hz to avoid charge build-up in the scala media. Custom Labview-based data acquisition software ensured that the exact phase both acoustic and electrical stimulus with respect to each individual pixel is known, making it possible to reconstruct the motion of the sensory cells using custom Matlab scripts. The software tracked the temporal relation between the pixels and the sound stimulus. Image sequences free from motion artefacts were then reconstructed using a Fourier series approach, to generate a sequence of 12 images at equally spaced phases of the sinae wave in Matlab. Each frame was specific for one phase of the stimulus. Images for positive and negative current stimulation were also reconstructed at 12 equally spaced phases. These image sequences were low-pass filtered and subjected to wavelet-based optical flow analysis.^20^ To improve the signal-to-noise ratio, trajectories for all pixels in a 5 × 5 region were averaged. The pixel size was adjusted to allow measurement of motions down to ∼30 nm. When imaging bundle movements, each experiment began by acquiring a baseline of 2 sets of images over a period of 15 min. Once a stable response was verified, ATP was applied either via a brief pulse of pressure injection into the scala media or perfused through scala vestibuli across basilar membrane.

### ATP perfusion, injection and acoustic overstimulation

For experiments in which multiple solutions (such as ATP, TNP-ATP or Yo-Pro-1) were injected into the endolymphatic space after fluorescent staining with di-3-anappdhq, the first microelectrode was gently retracted with the hydraulic motor and removed from the apical opening. A second microelectrode containing 5 mM ATP dissolved in artificial endolymph was inserted at a location close to the opening made in the Reissner's membrane by the first electrode. Pipettes were positioned 50–70 μm from the hair bundles. ATP was pressure-injected at 12 psi for 5–10 s. To verify the injection worked, a time series of confocal images, 60 s in length, was acquired during each injection. Cochlear microphonic potentials (CM) were recorded before and at 5-min intervals after the injection for 30 min. Sets of confocal images for the determination of hair bundle displacement were recorded before (2 sets) and after injection (2 sets) during the following 30 min at a stimulus level of 85 dB SPL and 75 dB SPL, 10 μA at 200 Hz best frequency.

For experiments in which solutions were perfused into the perilymphatic space after fluorescent staining with Calcein-AM, microphonic potentials were recorded every 2–3 min intervals before and followed with every 5 min intervals after the application and after washout. Sets of confocal images for hair bundle displacement determination were recorded before (2 sets) and after the ATP application (3 sets), and after the washout (3 sets) for the complete duration 70–80 min of the whole experiment at a stimulus level of 85 dB SPL and 75 dB SPL, 10 μA at 200 Hz best frequency.

For acoustic overstimulation experiments, preparations were exposed to tone bursts at 104 dB SPL, usually at 160 or 180 Hz, 20 Hz below the best frequency (corresponding to the peak of the tuning curve), for 10 min. Each tone burst lasted for 700 ms and were repeated at 1-s intervals. Microphonic potentials (at the stimulus frequency) were recorded before and after the overstimulation. Fluorescent images were recorded after the application of solutions, and immediately after the overstimulation, and after 10 min.

No sign of tissue damage such as stereocilia-TM decoupling, TM detachment, or swelling of the organ of Corti or TM was observed during ATP perfusion or injection procedure. The media was buffered to maintain pH. The change in osmolarity was 0.3% from 1 mM ATP perfusion and 0.15% from 5 mM ATP injection which was not a significant change.

### Confocal imaging

Samples were imaged with an upright laser scanning confocal microscope (Zeiss LSM 780) fitted with a 40x, 0.80 numerical aperture water immersion objective lens controlled with the ZEN 2012 software. Confocal images were obtained in two track channel mode or online finger printing mode with linear unmixing with excitation at 488 nm for Calcein-AM fluorescence and at 561 nm for di-3-aneppdhq fluorescence with MBS (488/561). For most preparations, Z-stacks were acquired at 12-bit pixel depth, 512 × 512 pixels, with an integration time of 6.30 μs per pixel, pinole of 1.0 Airy units and a spacing 1.0 or 3.0 μm section spacing per slice with 30–60 slices up to 100 μm in total depth. Fluorescent images were obtained before and after the application of ATP and acoustic overstimulation. 5 μM of Yo Pro-1 dye was injected into scala media and its uptake was determined to locate and identify P2X2 receptor protein in the structures of the organ of Corti. Images were processed in ImageJ 2.9.0/1.53t software, ZEN 2012, and Matlab (R2022b, the Mathworks, Natick, MA, USA).

The mean fluorescence intensity corresponding to each channel was automatically calculated in ZEN 3.1 based on the region of interests drawn. A randomly chosen area lacking specific signal was used to determine the background level, which was then subtracted from the respective ROIs in an individual image. Individual fluorescence intensity values of a given ROIs were normalised to the mean fluorescence intensity of Reissner's membrane epithelial cell of the corresponding preparations.

### Interferometry and confocal imaging

The right temporal bone was excised from the same animals used above or a different study; therefore, no additional animals were sacrificed to conduct these experiments. The preparation was mounted in a custom-built holder, and the microdissection was performed in the apical turn and base of the cochlea as described earlier. The holder with the dissected preparation was mounted at a custom-built displacement-sensitive interferometer with a calibrated loudspeaker, perfusion system and microelectrode in place. The system, equipped with an integrated Zeiss LSM Pascal confocal microscope and an objective lens with 25× magnification, was used for measuring acoustically and electrically evoked motion of the organ of Corti and to map morphological parameters.^18^ The system allows measurements of sound-evoked motion without the use of artificial reflectors as the cells in the organ of Corti (usually Hensen's cells) have sufficient optical reflectivity. The preparation was oriented with the basilar membrane perpendicular to the optical (transverse) axis. To assess the direction of motion, the steps taken are described elsewhere.^21^ The location of the measurement spot on the Hensen's cells was determined by imaging the focused measurement beam through the confocal microscope. Sound-evoked motion was measured in response to a 1-s acoustic stimulus with 10 frequency components ranging between 60 and 510 Hz. The stimulus was presented 10–20 times to allow averaging, at stimulus level of either 62 or 82 dB per frequency point. If the interferometer's carrier signal amplitude dropped, the measurement was automatically rejected by the Labview-based custom data acquisition software. In some experiments, acoustic stimulation was combined with electrical stimulation delivered through a microelectrode positioned in scala media in the apical turn. The electrical stimulus was a linear current ramp going from +5 μA to −5 μA over the course of 500 ms.

All sound pressures were corrected for attenuation caused by immersion of the preparation in tissue culture medium. The displacement from the signal at each stimulus frequency was extracted offline using custom Matlab scripts.

Confocal Z-stacks in a continuous time series mode were acquired at 8-bit pixel depth, 512 × 512 pixels, with an integration time of 12.8 μs per pixel, pinhole of 2.5 Airy units and with 15 slices up to 50–100 μm in total depth. The laser line at 488 nm with matching filter was used. Confocal image stacks were used for three-dimensional reconstructions for determining spatial relations using Matlab or Image J software's.

### Fluorescence correlation spectroscopy (FCS)

Microdissection and sample preparation were performed in similar way as described in Animal and experimental model details, but with slightly larger opening in the apex to enhance light passage. The holder was placed under the same confocal microscope setup as in Time-resolved rapid confocal imaging. Calcium indicator dye Calbryte 590 potassium salt (AAT Bioquest), dissolved in the endolymph-like solution at 50 nM concentration, was used to fill the microelectrode. In an FCS measurement, indicator molecules traverse the ∼1-fL detection volume of a confocal microscope through random diffusion. Once inside the detection volume, molecules were excited by a 561 nm laser beam and started emitting light. From the fluorescence fluctuations that occur when molecules enter or leave the detection volume, the average number of dye molecules and their calcium-dependent molecular brightness were obtained.

The solution was introduced from the microelectrode to scala media with gentle pressure injections (∼12–13 psi, Picospritzer II) until the fluorescence count rate in scala media reached the level of 70–100 kHz. The dye was let to diffuse for at least 3 min before recordings. Fluorescence signal was recorded at several recurring locations by targeting the excitation laser beam (561 nm) to the centre of the tectorial membrane and closer to the outer hair cells. In addition, the fluorescence signal was always recorded in scala media (endolymph) at ∼40–60 μm distance from the tectorial membrane both before and after the recordings from TM. The exact locations were selected based on a structural image obtained by reflection imaging. At each location, 10 s fluorescence recordings were repeated 10 times before (2 sets) and after ATP introduction (2 sets), and after ATP washout (2 sets). Further data analysis was performed in Matlab (MathWorks). The autocorrelation of the signal was calculated, and the resulting correlation curves were averaged over repetitions for each location. The data were normalised to before ATP where both repetitions were averaged and the second repetitions after ATP and washout in endolymph and TM locations were considered. Thereafter, three-dimensional anomalous diffusion model equation including the triplet state term^22^^,^^23^

1 G(t)=Neff−1[1+(tτdiff)α]−1(1+(tτdiff)α1S2)−12

was fitted to the correlation curve. In the equation, Neff is the average number of fluorescent molecules in the effective detection volume, t represents the time lag of autocorrelation, τ_diff_ is the average lateral diffusion time through the detection volume, α is the exponential parameter for anomalous diffusion, and S describes the shape of the detection volume. When appropriate, a term accounting for the triplet state formation was also included. This fitting enables the calculation of molecular brightness (mb) by dividing the average fluorescence count rate (cr) of the recordings by N_eff_. The molecular brightness of the calcium indicator dye depends on free calcium. The values of N_eff_ were corrected for the effect of background fluorescence as previously described.^22^ The background fluorescence level was recorded at each of the locations before inserting the microelectrode through the RM.

### Whole-cell patch clamp recordings

Electrophysiological recordings from Reissner's membrane epithelial cells were made as described. The left temporal bone was excised from young normal hearing guinea pigs, where right temporal bone from the same animals was used for another study; therefore, no additional animals were sacrificed to conduct these experiments. Microdissection was performed in the apical turn and base of the cochlea as described earlier. The apical opening was made slightly bigger in comparison to trapezoidal opening used for other *ex vivo* recordings. The dissected preparation was immersed in cell culture medium (DMEM) and was mounted at a Zeiss confocal LSM 780 with calibrated loudspeaker and perfusion system and with patch clamp recording and injection microelectrodes in place and maintained at room temperature.

Tight-seal whole-cell voltage clamp recordings were performed at the epithelial cells of the intact Reissner's membrane facing the perilymphatic space. Intracellular solution contained (in mM): 10 KCl, 0.1 CaCl_2_, 1 MgCl_2_, 1 EGTA, 10 Hepes, 120 K-gluconate, 4 Mg-ATP, 0.3 Na-GTP, pH 7.2^24^, ^25^, ^26^ combined with 0.2% Lucifer yellow dye in some preparations to visualise the recorded cell. Borosilicate glass pipettes had resistance of 4–7 MΩ and a liquid junction potential of −14 mV was corrected for. We applied a single sweep voltage clamp protocol lasting for 15–20 min at a holding potential of −60 mV. Voltage clamp step protocols were recorded before and after this single sweep protocol. One minute into the single sweep protocol, 1 mM ATP was added to the gravity driven perfusion for 3 min followed by washout.

For experiments in which 5 mM ATP was injected into the endolymphatic space, two recording pipettes were used simultaneously. One pipette was used for patch clamp recording and the other one for recording the cochlear microphonic potential and for injection of solutions into the scala media. While penetrating Reissner's membrane, care was taken not to overstretch the membrane. The first penetrating microelectrode with 2–3 μm tip diameter was positioned 60–80 μm from the hair bundles near the attachment of Reissner's membrane to the stria vascularis. The second electrode for patch clamp recording was positioned 80–100 μm closer to the centre of the Reissner's membrane and 200–250 μm upwards from the injection electrode. ATP was pressure-injected at 10 psi lasting for 5 s during the voltage clamp protocol. Microphonic potentials were recorded at a stimulus level of 85 dB SPL, 200 Hz best frequency before, during and after the voltage clamp protocol. Simultaneous patch imaging and confocal imaging in reflection mode were performed before, during and after the voltage clamp protocol. For most preparations, Z-stacks were acquired at 12-bit pixel depth, 512 × 512 pixels, with an integration time of 6.30 μs per pixel, pinhole of 2.0 Airy units and a spacing 3.0 μm per slice with 60–80 slices up to 250–300 μm in total depth. The laser line at 561 nm and matching beamsplitter was used.

The biophysical properties of Reissner's membrane epithelial cells were as follows: membrane capacitance (Cm) 15–50 pF; cell membrane resistance (Rm) 300 MΩ–3 GΩ; series resistance between 10 and 30 MΩ (n = 8) with outward current rectification evident positive to ∼ −60 mV in voltage clamp step protocols which differed from mouse Reissner's membrane mesenchymal cells which exhibited Cm 8–9 pF; Rm 600–800 MΩ; with outward rectification to ∼−30 mV in voltage ramps.^12^

Membrane voltages and currents were recorded with a MultiClamp 700B amplifier and a Digidata 1440A (Molecular Devices), controlled by pCLAMP 10.3 software (Molecular Devices), sampled at 5–10 kHz, and low pass filtered at 2–5 kHz. The data were analysed in pCLAMP 11.2.2 (Molecular Devices) and Matlab.

### Statistics and replicates

All experiments were repeated multiple times; the number of individual measurements and the number of preparations is included in the main body of the text and in the figure legends. No prior *ex vivo* animal studies examined sound-evoked responses, calcium changes, or used live-cell imaging in *ex vivo* models of perilymphatic ATP role in protection from loud-sound induced hearing loss. Hence, there was no data available to use for power calculations, and none were performed. In the *ex vivo* animal study Drs. Karlsson, Pitkänen, and Fridberger were aware of all the stages of different experimental protocols involving patch, FCS and interferometry data. Randomised group assignment was not used but animals were supplied from the same breeder, they were of the same species, weight and age range, housed under identical conditions and received the same food, which minimised common sources of variability.

Bundle displacements and hearing organ vibration data and mechanics were analysed using custom written Matlab scripts as described in.^18^^,^^19^ Statistical tests were performed using GraphPad Prism 10. Plots were generated in Prism software's and Matlab. Normality and Lognormality tests (including Kolmogorov-Smirnov test) was used to assess the distribution. Data before and after on the same preparation were analysed using the parametric ratio paired *t*-test for normally distributed data and non-parametric Wilcoxon matched pairs signed-rank test for non-normally distributed data as appropriate. Data before, after ATP and after washout on the same preparation were analysed using RM (repeated measures) or REML (Mixed-effects model) one-way ANOVA followed with post-hoc testing Tukey's multiple comparisons with Geisser-Greenhouse's correction for normally distributed continuous data (parametric). Kruskal-Wallis followed with post-hoc testing Dunn's multiple comparisons for non-normally distributed continuous data (non-parametric) as appropriate. Ordinary one-way ANOVA with post-hoc testing Tukey's multiple comparisons was used for parametric data having separate groups >2. For non-parametric paired data Hodges–Lehmann estimator was used for calculating median difference and 95% CI. Mean and median difference was calculated between (ATP-Before) and (Washout-ATP). All statistical tests were two-tailed, and significance was assigned at *P* < 0.05. Details of the statistical tests used in each case were given in the results text.

### Role of funders

Funders were not involved in design, data collection, analyses, interpretation or writing.

## Results

What is the precise cellular and molecular mechanism by which extracellular ATP, acting across the Reissner's membrane barrier, exerts a protective effect on cochlear function during loud sound exposure? To answer this question, we used temporal bone preparations isolated from guinea pigs to investigate the physiological and mechanistic role of ATP in the inner ear upon loud sound exposure.

A total of 108 animals were used for ATP and TNP-ATP perfusion and injection experiments. 39 were excluded due to middle-ear infection, dissection damage, or technical issues. Of the remaining 69 preparations, 36 were used for ATP perfusion experiments, with 8 later discarded because of damage or incomplete recordings. 19 preparations were used for TNP-ATP experiments, of which 6 datasets were lost due to technical issues.

### ATP receptor localisation and structural changes following overstimulation

We began by verifying the distribution of ATP receptors and its localisation in the cochlea via live staining by using a DNA-binding fluorescent dye, Yo-Pro-1, delivered via injection into the endolymphatic space (scala media). The dye-stained cation-selective, plasma-membrane P2X receptor expressing cells under physiological conditions, showing a pattern partly consistent with that observed in mouse, rat, and guinea pig inner ears.^27^, ^28^, ^29^

**Fig. 1:**
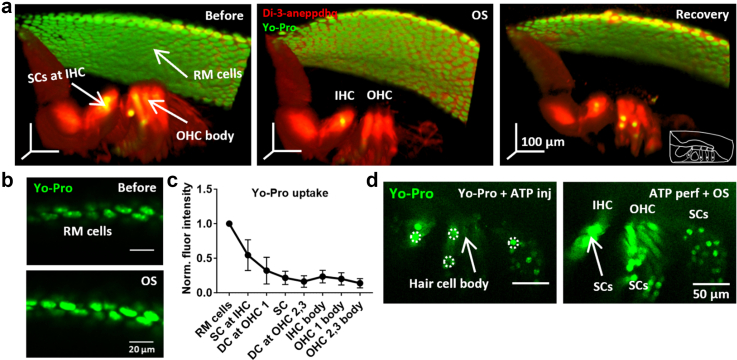
**ATP receptors and structural change upon overstimulation and ATP in the organ of Corti.** (**A**) 3D reconstruction of confocal image stacks of the guinea pig cochlear organ of Corti showed structural and fluorescence intensity change in the Reissner's membrane (RM), hair cells and supporting cells (SCs) before and after overstimulation (OS) identified using red membrane dye di-3-ANEPPDHQ and green dye Yo-Pro-1. Inset in white showed the schematic view of the organ of Corti (OoC). The acoustic stimulus used was a 160-Hz continuous tone burst at 104 dB SPL for 10 min. (**B**) Increase in the Yo Pro dye uptake was observed in the RM epithelial cells after OS. No change in the Yo Pro dye uptake observed in the hair cells and supporting cells after OS. (**C**) Normalised mean ± s.d. of fluorescence intensity profiles representing Yo-Pro-1 dye uptake in the cochlea structures (15 preparations): RM epithelial cells strongly expressed ATP receptors followed with SC, IHC and OHC 1st row cell bodies. No labelling was observed on the hair cell stereocilia or any other cell types. (**D**) Dye uptake increased in the hair cells when ATP was perfused followed with OS indicating enhanced P2X2 (ATP-gated) receptor activation via 1 mM ATP perfusion and not 5 mM ATP injection or OS alone. White dotted circles represented the hair cell body, supporting cells at IHC and OHC and supporting cells.

The fluorescence staining was strongest in Reissner's membrane (RM) epithelial cells and supporting cells (SCs) with faint staining in hair cells (Fig. 1A). Using caged ATP activation together with Yo-Pro-1 staining, the presence of ATP receptors in cochlear cells was confirmed (Supplemental Fig. S1A). Sustained loud sound (10 min at 104 dB SPL) led to bulging of RM and contraction of the hearing organ followed by recovery over the following 10–15 min, as seen in three-dimensional reconstructions (Fig. 1A). The overstimulation (OS) produced enhanced dye loading in RM cells shown through increased fluorescence intensity, but not in supporting cells or hair cells (Fig. 1B). To quantify the dye uptake, the fluorescence intensity across cochlear regions was measured. The fluorescence profiles (Fig. 1C, n = 15) confirmed higher uptake of the dye in the RM epithelial cells followed with supporting cells, and hair cell bodies in healthy preparations. Perfusion of 1 mM ATP in the perilymphatic space together with overstimulation enhanced Yo-Pro-1 staining in SCs and hair cells bodies regions (Fig. 1D) and alteration of RM in some preparations. There was no enhanced staining with ATP injection or overstimulation alone. The enhanced ATP receptor activation, indicating ATP-gated pore formation and associated structural changes during overstimulation, prompted us for closer examination of sound-evoked responses.

### Perilymphatic ATP decreased sound-evoked potentials

Having established that the ATP receptors are located in RM cells, supporting cells and hair cells, we proceeded to record sound-evoked electrical potentials. During sound stimulation, ions permeate mechano-transduction (MET) channels from the surrounding fluid, generating extracellular electrical and endocochlear potentials, which can be measured through the electrode placed near the sensory cells. The electrical potentials have a rapidly varying component that follows the frequency of the sound stimulus, the cochlear microphonic potential, (CM) and a DC component, the summating potential (SP). Tuning curves were acquired by tracking the amplitude of these potentials over a range of stimulus frequencies. Concurrently with recordings, ATP was administered via perfusion into the perilymph or by injection into the endolymph.

**Fig. 2:**
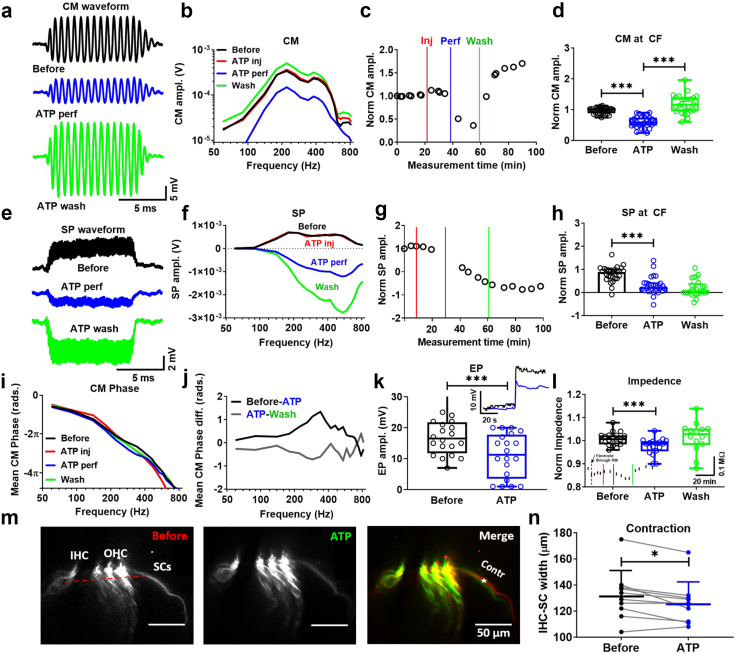
**ATP in scala vestibuli decreased cochlear microphonics and summating potentials.** (**A**) Waveforms of the cochlear microphonic potential (CM) before ATP (top), after perfusion of 1 mM ATP dissolved in clear medium (centre), and after washout of ATP (bottom). (**B**) CM tuning curves before, during ATP perfusion, after ATP injection and after the washout of ATP in an example preparation (different experiment than A). Note no change after ATP injection (red), the decrease after ATP perfusion (blue), and full recovery after washout (green). (**C**) Time course of the CM peak amplitude normalised to the amplitude recorded before ATP application at the start of the experiment (same experiment as in B). ATP perfusion evoked a fast reduction within 2–3 min and rapid recovery within the next 45–50 min following the washout. The vertical lines indicates the time of injection, perfusion and washout of ATP. (**D**) Averaged CM amplitude response at characteristic frequency (CF) shows a significant reduction after ATP and recovery above the baseline after washout (n = 28). (**E**) Waveforms of the SP before ATP (top), after ATP introduction (centre), and after washout of ATP (bottom) (different experiment than in A). (**F**) SP curves before, after ATP and after the washout in an example preparation (different experiment than in E). Note the decrease and flip of polarity after ATP perfusion and continued decrease after washout. (**G**) Time course of the normalised SP peak amplitude displayed a fast reduction within 2–3 min after ATP introduction with continued reduction following the washout. The vertical line indicates the time of perfusion and washout of ATP (same experiments as in F). (**H**) Averaged SP amplitude response at CF showed a significant reduction after ATP and after the washout (n = 26). Data is the median ± IQR. (**I**) Phases of the CM measured relative to the voltage driving the loudspeaker. Note the phase lag after ATP perfusion and recovery after washout. (**J**) Mean phase difference between before, after ATP and after the washout, excluding preparations where the CM was close to the noise floor of the recording. (**K**) Averaged EP amplitude recorded at the start and end of the experiment showed a significant reduction after ATP (n = 20). Inset figure showed the EP amplitude before and after ATP. (**L**) Averaged impedance recorded throughout the experiment showed a significant reduction after ATP (n = 18). Inset figure showed the impedance drop after ATP. All data sets were normalised to the initial value recorded before the ATP perfusion. The acoustic stimulus was a 200-Hz tone burst at 75–80 dB SPL. Data are the means ± SD. ∗∗∗*P* < 0.001; ∗*P* < 0.05; n.s., not significant; REML and Kruskal-Wallis one-way ANOVA followed with post-hoc tests and Paired *t*-test applied as appropriate. (**M**) Confocal images using red membrane dye di-3-ANEPPDHQ showed morphological change at supporting cells level when ATP was perfused (red = before, green = after; white star). To determine the change in the morphology, the width from the IHC nucleus to the SCs boundary was measured. (**N**) ATP perfusion led to the contraction of SCs across several preparations (n = 10) indicated structural changes in the organ of Corti.

Perfusion of 1 mM ATP led to a pronounced decline in the amplitude of the CM. The decline was followed by recovery above the baseline, as seen in the example waveforms and tuning curves in Fig. 2A and B (stimulus level 80 dB SPL). The drop in the CM amplitude was evident 2–3 min after ATP introduction and remained during the ensuing 15–20 min (Fig. 2C). CM started to recover rapidly after ATP washout with amplitude larger compared to baseline during the ensuing 35–40 min (Fig. 2C). On average, the CM peak amplitude decreased from 486 ± 475 μV to 315 ± 362 μV after ATP introduction (Fig. 2D; *P* < 0.001, REML one-way ANOVA, mean difference −170 μV, CI −231 to −109, *F*_*1, 37*_ = 57, Tukey's multiple comparisons test; n = 28) and increased to 556 ± 567 μV after the ATP washout (Fig. 2D; *P* < 0.001, REML one-way ANOVA, mean difference 217 μV, CI 130–304, Tukey's multiple comparisons test; n = 25) at characteristic frequency of 180 Hz.

In 21 out of 28 preparations, 5 mM ATP in combination with 5 μM Yo-Pro-1 was injected in scala media either before or during the perilymphatic ATP perfusion. Interestingly, ATP injection failed to alter the microphonic electrical potentials (red trace, Fig. 2B and C), which provided indirect indication that ATP present in endolymph exerted no measurable effect.

The SP can be positive or negative depending on stimulus level and frequency. At 80 dB SPL, the SP flipped its polarity after introducing ATP into perfusion as shown in example waveforms and curves (Fig. 2E and F). The drop in the SP peak amplitude was evident 2–3 min after ATP introduction and the decline remained for 35–40 min after ATP washout (Fig. 2G). The change in SP caused by ATP introduction was significant, on average from (median −27 μV, iqr 197) to (median −66 μV, iqr 159) (Fig. 2H; *P* < 0.001, Kruskal-Wallis one-way ANOVA, median difference −30 μV, CI −61 to −11, Dunn's multiple comparisons test; n = 27) at characteristic frequency of 180 Hz. Further decrease (median −180 μV, iqr 528) occurred after ATP washout (Fig. 2H; *P* = 0.53, Kruskal-Wallis one-way ANOVA, median difference −284 μV, CI −531 to −121, Dunn's multiple comparisons test; n = 25). The timing of the sound-evoked response shifted after ATP introduction and washout (Fig. 2I; n = 28), as seen in the phase of the cochlear electrical potential plotted with respect to the voltage driving the loudspeaker. This revealed a slight phase lag after ATP introduction (Fig. 2J; n = 28) present from 200 Hz up to 700 Hz.

Similar recordings were made at moderate sound pressure level 65–70 dB SPL (Supplemental Fig. S1B–G) in the same preparations as in Fig. 2.

To conclude, at moderate high sound level, extracellular ATP in perilymph had an acute and reversible modulation of cochlear electrical potentials which may reflect a short-term protection.

The endocochlear potential (EP) and impedance inside scala media were measured to examine the ionic alterations in the cochlea. The absolute values of the EP in the beginning and in the end of experiment were compared. EP decreased significantly from 17 ± 6 mV to 11 ± 7 mV, (Fig. 2K; *P* < 0.001, mean difference −6 mV, CI −9 to −3, *t* 4, *df* 19, paired *t*-test; n = 20) seen in an example (Inset, EP curve declined after ATP perfusion). The impedance (from electrode and preparation) was measured inside the scala media throughout the time frame of ATP pre-perfusion, perfusion and washout. The impedance decreased significantly from 7.5 ± 1.6 MΩ to 7.2 ± 1.6 MΩ (Fig. 2L; *P* < 0.001, REML one-way ANOVA, mean difference −0.3 MΩ, *F*_*1,*__*17*_ = 4, Tukey's multiple comparisons test; n = 18) after ATP and recovered to baseline (Fig. 2L; *P* = 0.1, REML one-way ANOVA, Tukey's multiple comparisons test; n = 14) after ATP washout as seen in an example preparation (Inset, Impedance altered after ATP and recovered to normal level). The alterations in EP and impedance were consistent with the changes in CM and SP.

The structural changes of the hearing organ after perfusing ATP into perilymph was quantified by measuring the OoC width from the IHC nucleus to the SCs boundary (Fig. 2M and N). ATP led to contraction from 132 ± 18 μm to 126 ± 16 μm (Fig. 2N; *P* = 0.01, mean difference −6 μm, CI −10 to −2, *t* 3, *df* 19, Paired *t*-test; n = 10). Thus, ATP in perilymph suppressed EP and impedance and led to structural alteration of the OoC indicating indirect effects towards protection.

### Perilymphatic ATP decreased outer hair stereocilia deflections

To evaluate whether the reduction in electrical responsiveness was caused by an altered stimulus delivered to stereocilia, we examined the sound-evoked responses of stereocilia using time-resolved confocal imaging. Stereocilia bundles were labelled with the fluorescent dye di-3-ANEPPDHQ, which was delivered through the same microelectrode used to record electrical responses near the sensory cells.

**Fig. 3:**
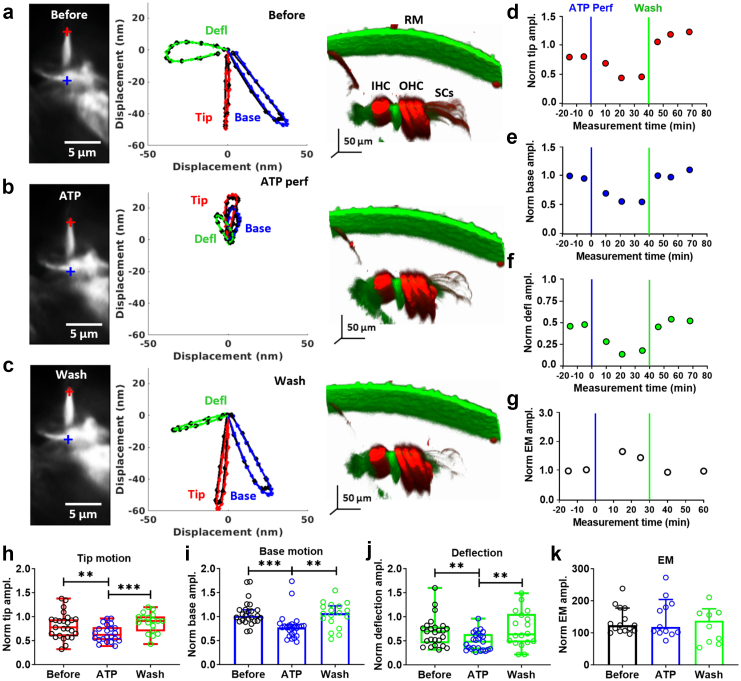
**ATP in scala vestibuli induced reduced sound-evoked motion of outer hair cell stereocilia.** (**A–C**) Time-resolved confocal images using red membrane dye di-3-ANEPPDHQ acquired during sound stimulation show that ATP does not alter the morphology of outer hair cell stereocilia (except for a small change in the brightness of the fluorescent dye). Sound-evoked motion trajectories of the stereocilia tip (red) and base (blue) before and after 1 mM ATP perfusion and after the washout in an example preparation. Note the reduction in the tip motion (red) and base motion (blue) leading to decreased deflection (green) after ATP perfusion. 3D-reconstructions of confocal image stacks obtained in the same preparations, showed a fainter staining and slight contraction of supporting cells (green = Calcein-AM, red = Di-3-ANEPPDHQ; 2nd row, third panel). (**D–F**) Time course of the tip motion amplitude (red circle), base motion amplitude (blue circle) and deflection amplitude (green circle) of stereocilia of preparations shown in (A–C). The vertical line at time zero indicates the time of ATP application and the later line the time of washout. Data is normalised to the trajectory amplitude recorded before ATP application. (**G**) Time course of the electromotility before and after ATP, and washout. The vertical line at time zero indicates the time of ATP application. Data normalised to the amplitude recorded before ATP perfusion. (**H–J**) Averaged motion at the base (blue bar), tip (red bar) and the deflection (green bar) of stereocilia showed significant decrease after ATP perfusion. Data were normalised to the initial base trajectory amplitude recorded before the ATP perfusion (n = 16). Data are the means ± SD. (**K**) Averaged electromotility amplitude showed no significant change after ATP (n = 15). The acoustic stimulus was a 200 Hz tone burst at 75–80 dB SPL with ±10 μA. Data is the median ± IQR. ∗∗∗*P* < 0.001; ∗∗*P* < 0.01; n.s., not significant; REML and Kruskal-Wallis one-way ANOVA followed with post-hoc tests applied as appropriate.

1-mM ATP was added to the perfusion solution. No morphological changes were observed in stereocilia (except for minor alterations in the brightness of the dye, Fig. 3A–C). As seen in the example data in Fig. 3A–C the sound-evoked displacement at both the tip of the stereocilia (red trajectory) and their base (blue trajectory) significantly decreased after ATP introduction and following ATP washout, the tip and base motion amplitude recovered during acoustic stimulation at 80 dB SPL and 200 Hz. The decreased motion at the tip and base after ATP led to a reduced deflection amplitude (green trajectory) (Fig. 3D and E). The reduction in the deflection amplitude was apparent 2–5 min after ATP introduction and the amplitude continued to be reduced for at least 10 min, until a rapid recovery was seen after ATP washout exceeding the baseline over the ensuing 45–50 min (Fig. 3F). Aggregated data across 16 preparations are shown in Fig. 3H–J. The decrease in motion amplitude at the base of stereocilia was significant from (median 126 nm, iqr 36) to (median 92 nm, iqr 45) (Fig. 3I; *P* < 0.001, Kruskal-Wallis one-way ANOVA, median difference −41 nm, CI −59 to −33 nm, Dunn's multiple comparisons test) as was the change in displacement at their tips from 102 ± 33 nm to 82 ± 21 nm (Fig. 3H; *P* = 0.009, REML one-way ANOVA, mean difference −16 nm, CI −29 to −4 nm, *F*_*2,*__*35*_ = 9, Tukey's multiple comparisons test) after ATP introduction. The deflection amplitude decreased significantly from 85 ± 38 nm to 62 ± 23 nm (Fig. 3J; *P* = 0.003, REML one-way ANOVA, mean difference −25 nm, CI −39 to −11 nm, *F*_*2,*__*39*_ = 9, Tukey's multiple comparisons test). After ATP washout the base motion amplitude recovered to baseline (median 101 nm, iqr 75) (Fig. 3I; *P* = 0.002, Kruskal-Wallis one-way ANOVA, median difference −13 nm, CI −36 to6 nm, Dunn's multiple comparisons test). The tips displacement increased significantly to 109 ± 25 nm (Fig. 3H; *P* < 0.001, mean difference 31 nm, CI 13–49 nm, REML one-way ANOVA, mean difference −27 nm, Tukey's multiple comparisons test) with amplitude larger than baseline. Similarly, the deflection amplitude increased significantly to 93 ± 46 nm (Fig. 3J; *P* = 0.001, REML one-way ANOVA, mean difference −25 nm, CI 8–41 nm, Tukey's multiple comparisons test).

Along with these recordings, we acquired Z-stacks of confocal images and performed 3D reconstructions to visualise structural changes. The 3D reconstructions revealed a small contraction at supporting cells level after ATP introduction (Fig. 3A–C, right most panels, corresponds to the observation presented in Fig. 2M and N).

Stereocilia motion recordings were also performed at moderate sound pressure level 70 dB SPL (Supplemental Fig. S1H–J) after ATP introduction in the same preparations as shown in Fig. 3. No alteration in the overall OHC bundle deflection was detected.

To conclude, extracellular ATP perfused in scala tympani entering scala vestibuli through helicotrema had an acute and reversible suppression effect of the stereocilia deflections at moderate high sound level contributing towards a short-term cochlear protection against moderately loud sound.

The outer hair cell electromotility is critical for hearing, and to probe ATP's influence on hair cell function, we measured electrically evoked cell motility using the time-resolved rapid confocal imaging technique. The microelectrode allowed us to apply 10-μA square wave currents, which changes the electrical potential in scala media, resulting in increased currents through the MET channels and increased force production by outer hair cells. The electromotility amplitude changes were quantified through optical flow analysis. Perilymphatic ATP perfusion led to a minor increase in the electrically evoked motility. The change in the electromotility started 2–5 min after the application of ATP and reversed to baseline 20–25 min after washout (Fig. 3G). The amplitude altered ∼25–30 nm, but the change was non-significant from (median 123 nm, iqr 71) to (median 118 nm, iqr 103) (Fig. 3K; *P* > 0.99, Kruskal-Wallis one-way ANOVA, median difference 4 nm, CI −7 to55 nm, Dunn's multiple comparisons test; n = 15). There was a slow recovery to baseline after ATP washout to baseline (median 137 nm, iqr 105) (Fig. 3K; *P* > 0.99, Kruskal-Wallis one-way ANOVA, median difference −18 nm, CI −71 to −6 nm, Dunn's multiple comparisons test). No change in the electromotility was observed at low sound level (Supplemental Fig. S1K). The high variability observed suggests that OHC electromotility has an intrinsic component and highlights the limitations of obtaining direct measurements under these experimental conditions. By modulating the mechanical amplification (2) consistent with the decreased stereocilia stiffness after ATP the OHC may appear to play an indirect role towards a protective outcome.

### Endolymphatic ATP did not affect sound-evoked potentials

**Fig. 4:**
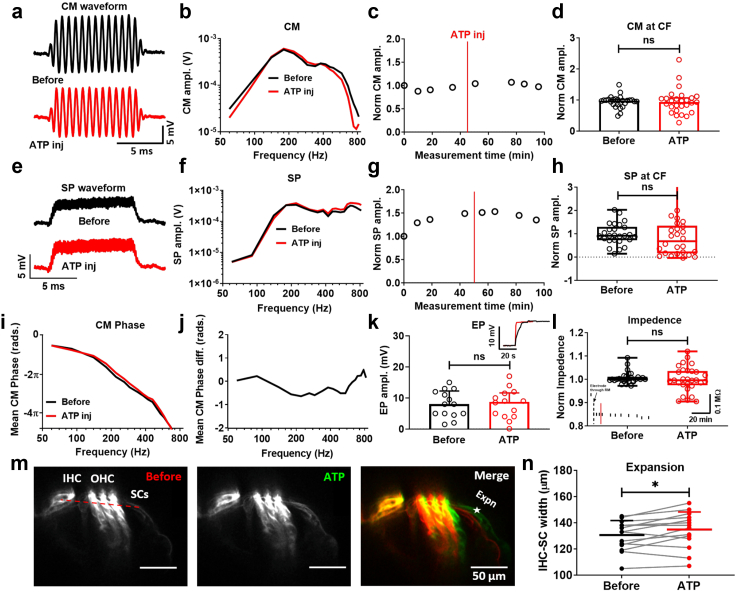
**No ATP effect on the sound-evoked electrical responses in scala media.** (**A**) Waveforms of the cochlear microphonic potential (CM) before ATP (left), and after injection of 5 mM ATP dissolved in normal endolymph (right). Each record represents a mean of 10 stimulus presentations. (**B**) CM tuning curves at CF before and after ATP injection in an example preparation. Each curve was recorded by stepping the stimulus frequency from 60 to 800 Hz, with 10 recordings averaged at each frequency (different experiment than A). (**C**) Time course of the CM peak amplitude normalised to the amplitude recorded before ATP injection at the start of the experiment. (**D**) Average CM amplitude response at CF showed no significant change after ATP injection (n = 27). (**E**) Waveforms of the summating potential (SP) before (left), and after injection of ATP (right) (same preparation as in A). Data is the median ± IQR. (**F**) SP curves before and after injection of ATP in an example preparation (different experiment than in E). (**G**) Time course of the normalised SP peak amplitude after injection of ATP. The vertical line indicates the time of injection. (**H**) Averaged SP amplitude response at CF showed no significant change after ATP injection (n = 26). Data is the means ± SD. (**I**) Phase of the CM measured relative to the voltage driving the loudspeaker. Note the slight phase lead after ATP injection. (**J**) Mean phase difference between before and after ATP, excluding preparations where the CM was close to the noise floor of the recording. (**K**) Averaged endocochlear potential (EP) amplitude recorded at the start and in end of the experiment showed no significant change after ATP injection (n = 14, Inset of example preparation). Data is the median ± IQR. (**L**) Averaged impedance recorded throughout the experiment showed no change after ATP injection (n = 28, Inset of an example experiment). All data sets were normalised to the initial value recorded before ATP injection. The acoustic stimulus was a 200 Hz tone burst at 75–80 dB SPL. Data is the means ± SD. ∗*P* < 0.05; n.s., not significant; Paired *t*-test and Wilcoxon signed-rank test applied as appropriate. (**M**) Confocal images using red membrane dye di-3-ANEPPDHQ showed morphological change at supporting cells level when ATP was injected into scala media (red = before, green = after; white star). To determine the change in the morphology, the width from the IHC nucleus to the SCs boundary was measured. (**N**) ATP injection led to expansion of SCs across several preparations (n = 14) indicated structural changes in the organ of Corti. Data is the means ± SD.

Having established that the ATP introduced in the perilymphatic compartment had a suppressive effect on the cochlear responsiveness, we investigated the role of ATP in the endolymphatic compartment. Artificial endolymph containing 5 mM ATP was applied to the endolymphatic space via injection (note that the effective concentration was lower because the injected solution was dissolved in the endolymph of scala media). For ATP injection experiment 27 preparations were used. Out of 27 preparations, 14 were used in the experiments described above, with ATP injection performed first, followed by ATP perfusion. At stimulus level of 80 dB SPL, ATP injection had no effect on the cochlear sound-evoked potentials as shown in waveforms and tuning curves (Fig. 4A and B). After ATP injection the amplitude remained unchanged during the ensuing 45–50 min (Fig. 4C). On average, the peak CM amplitude showed non-significant change from (median 581 μV, iqr 850) to (median 607 μV, iqr 930), measured at the peak of each tuning curve near 200 Hz (Fig. 4D; *P* = 0.5, median difference −3 μV, CI −22 to −21, W −58, Wilcoxon signed-rank test; n = 27).

The SP amplitude displayed in waveforms and curves (Fig. 4E and F) showed a minor decrease during the ensuing 45–50 min (Fig. 4G). On average, the SP amplitude changed from 87 ± 315 μV to −112 ± 196 μV, measured at the peak of each tuning curve (Fig. 4H; *P* = 0.2, mean difference −200 μV, CI −291 to −107, *t* 1, *df* 25, Paired *t-*test; n = 26). There was no significant change in the CM phase, showing a slight phase lead after ATP in comparison to before (Fig. 4I and J; n = 27). No change in the EP from (median 8 mV, iqr 7) to (median 9 mV, iqr 7.5), (Fig. 4K; *P* = 0.637, median difference −0.5 mV, CI −1 to2, W −16, Wilcoxon signed-rank test; n = 14) and no change in impedance from 8.2 ± 1.7 MΩ to 8.1 ± 1.3 MΩ (Fig. 4L; *P* = 0.74; mean difference 0.1 MΩ, *t* 0.3, *df* 24, Paired *t-*test; n = 29) was observed.

No change in staining of RM cells and Dieter cells after ATP injection was observed under fluorescence imaging (Supplemental Fig. S2A). Corresponding recordings at a moderate sound pressure level 70 dB SPL in the same preparations were performed (Supplemental Fig. S2B–G) as in Fig. 4. No changes were observed in the electrical potentials.

The structural changes of the hearing organ after injecting ATP into scala media were quantified by measuring the OoC width from the IHC nucleus to the SCs boundary (Fig. 4M and N). ATP injection led to expansion from 130 ± 11 μm to 134 ± 13 μm in SCs and across hair cells regions (Fig. 4N; *P* = 0.02, mean difference 4 μm, CI 0.7–8, *t* 2, *df* 13, Paired *t-*test; n = 14).

Endolymphatic ATP failed to modulate cochlear electrical potentials, indicating that it does not contribute to a protective effect.

### Endolymphatic ATP did not affect outer hair stereocilia deflections

Thereafter, the effects of ATP injection on sound-evoked motion of stereocilia were examined in the same preparations. After retracting the first microelectrode used for labelling the stereocilia with fluorescent dye di-3-ANEPPDHQ, ATP was injected close to the sensory cells in the endolymph through a second microelectrode containing 5 mM ATP in artificial endolymph (see Methods).

**Fig. 5:**
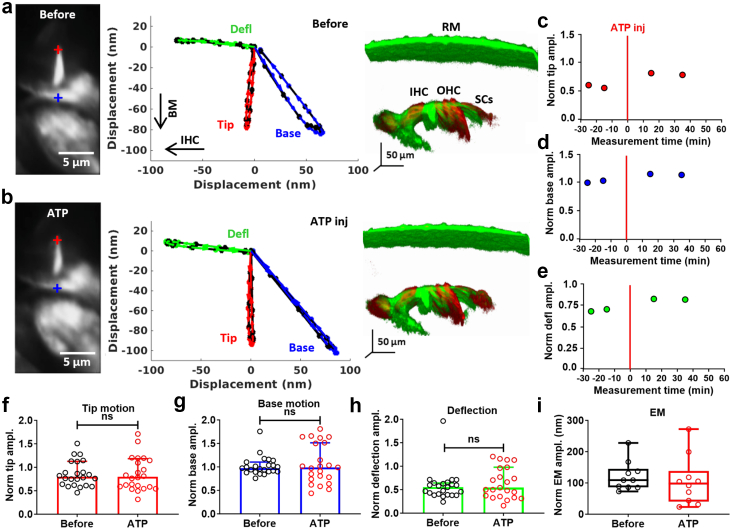
**No ATP effect on the sound-evoked outer hair cell stereocilia motion in scala media.** (**A, B**) Time-resolved confocal image using red membrane dye staining di-3-ANEPPDHQ of an OHC stereocilia bundle showed that the morphology is intact before and after the 5 mM ATP injection. Representative trajectories show no change in sound-evoked motion of the bundle tip (red) and base (blue) after ATP injection. 3D-reconstructions of confocal image stacks obtained in preparations (green = Calcein-AM, red = Di-3-ANEPPDHQ) with ATP injection, showed a little expansion of supporting cells (2nd row, third pnael). (**C–E**) Time course of the tip motion amplitude (red circle), base motion amplitude (blue circle) and deflection amplitude (green circle) of stereocilia in the preparations shown in (A–B). Data is normalised to the trajectory amplitude recorded before ATP injection. The vertical line at time zero indicates the ATP application. (**F–H**) Averaged motion at the base (blue bar), tip (red bar) and the deflection (green bar) of stereocilia showed no change after ATP injection (n = 12). Data were normalised to the base trajectory amplitude recorded before the ATP injection. Data are the median ± IQR. (**I**) Averaged electromotility amplitude showed no change after ATP injection (n = 12). All data sets were normalised to the initial value recorded before injection. Data is the means ± SD. The acoustic stimulus was a 200 Hz tone burst at 75–80 dB SPL with ±10 μA. n.s., not significant; Wilcoxon signed-rank test and Paired *t*-test applied as appropriate.

After the ATP injection, no morphological changes were observed in stereocilia (Fig. 5A and B). The injection did not change the response to a sound stimulus of 80 dB SPL at ∼200 Hz. As seen in the example data in Fig. 5A and B the sound-evoked displacement at both the tip (red trajectory) and the base (blue trajectory) of the stereocilia increased non-significantly after ATP. This minor transient increase was apparent 5–10 min after the injection and led to slightly increased deflection (green trajectory in Fig. 5B). The shape of the motion trajectories remained the same (Fig. 5A and B). 3D reconstruction of images showed a minor expansion after ATP injection (Fig. 5A and B) as displayed earlier in Fig. 3M. The slight increase in the deflection amplitude was apparent 5–10 min after ATP injection and the amplitude continued to be the same for at least 15–20 min, over the ensuing 40 min (Fig. 5F). The same phenomenon was observed for motion of the tip and base of stereocilia (Fig. 5D and E). Fig. 5H shows the stereocilia motion data across 12 preparations. At both the tip and the base, the motion amplitude non-significantly and transiently altered from (median 126 nm, iqr 3) to (median 134 nm, iqr 96) at the base (Fig. 5F; *P* = 0.916, median difference −8 nm, CI −39 to19 nm, W 8, Wilcoxon signed-rank test) and from (median 102 nm, iqr 64) to (median 104 nm, iqr 82) at the tip (Fig. 5G; *P* = 0.988, median difference −7 nm, CI −25 to28 nm, W −2, Wilcoxon signed-rank test). The deflection amplitude increased non-significantly from (median 71 nm, iqr 45) to (median 86 nm, iqr 91) (Fig. 5H; *P* = 0.142, median difference 7 nm, CI −6 to65 nm, W 98, Wilcoxon signed-rank test). Furthermore, we examined ATP's effect on electrically evoked motility of the outer hair cell. ATP caused no significant change in electromotility amplitude from 121 ± 47 nm to 106 ± 77 nm (Fig. 5I; *P* = 0.59, mean difference −16 nm, CI −79 to48 nm, *t* 0.5, *df* 9, Paired *t*-test; n = 10).

Endolymphatic ATP did not alter the OHC stereocilia motion and electromotility at moderate sound pressure level 70 dB SPL (Supplemental Fig. S2H–J, K) in recordings performed in the same preparations as in Fig. 5.

To conclude, the endolymphatic ATP did not modulate cochlear mechanics towards any sound pressure levels, which provides clear evidence that predictions of the cation-shunt model mechanism was not precise and hence not pertinent for short - term protection.

### Perilymphatic ATP decreased the organ of Corti motion

These cochlear mechanical findings diverged from those expected from the cation-shunt model. To validate this deviation, we applied an independent method and confirmed the results using laser interferometry on guinea pig temporal bone preparations.^18^ A total of 97 animals were used for the interferometry ATP-perfusion experiments: 62 at low sound levels (62 dB SPL) and 35 at high levels (82 dB SPL). At high levels, 21 of 35 preparations were used for Hensen's cell recordings; 18 of these were also used for cochlear microphonics, and electromotility was measured in 12 (2 excluded for technical issues). At low levels, 31 of 62 preparations yielded useable basilar-membrane and microphonic data; the remaining 31 were discarded due to technical problems. Of the 31 useable preparations, 6 were excluded from basilar-motion statistics and 10 from electromotility analysis. For ATP-injection experiments, 34 animals were used; 11 contributed to ATP basilar-motion statistics and 6 served as controls, while 17 were excluded due to incomplete or damaged preparations.

**Fig. 6:**
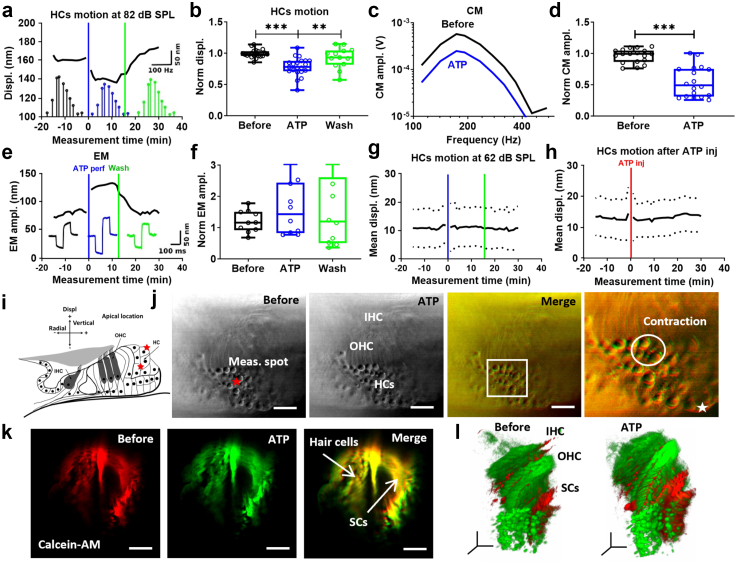
**ATP decreases organ of Corti motion at Hensen's cells level determined using laser interferometry.** (**A**) In an example experiment, the peak Hensen's cell (HCs) vibration motion amplitudes per frequency point changed noticeably after 1 mM ATP perfusion at a stimulus level of 82 dB SPL. The displacement amplitude went down after ATP and recovered back to baseline after washout (Inset). (**B**) Averaged data from (A) showed decreased displacement after ATP and recovery after washout (n = 21). (**C**) Example of cochlear microphonic tuning curves before and after ATP. Stimulus level, 82 dB SPL. (**D**) Average value of normalised microphonics amplitude displaying decrease after ATP (n = 18). (**E**) Current-evoked motility measured near the Hensen's cell site increased after ATP and recovered to baseline after washout during electrical stimulation. Current ramps going from −10 to +10 μA showed the increase in the amplitude (Inset). (**F**) Averaged electromotility (EM) amplitude showed amplification in the current-evoked motility after ATP (n = 10). All data sets were normalised to the value recorded before ATP perfusion. (**G**) Averaged interpolated data of the motion amplitude remained unchanged after ATP perfusion (blue line) at 62 dB SPL (n = 25), showed that the entire organ of Corti mechanics at Hensen's cells level works in a similar manner than the single hair cell stereocilia displacement at low sound level. Data is the median ± IQR. (**H**) Averaged interpolated data of the motion amplitude remained unchanged after 5 mM ATP injection (red line) at 72 dB SPL (n = 11). The vertical lines across the data indicate the time of ATP application at time zero followed with washout. Displ, displacement. Data are the means ± SD. ∗∗∗*P* < 0.001; ∗∗*P* < 0.01; n.s., not significant; RM, REML and Kruskal-Wallis one-way ANOVA followed with post-hoc tests, Paired *t*-test and Wilcoxon signed-rank test applied as appropriate. (**I**) A schematic view of the orientation and measurement spot (red star) in the recordings. (**J**) A shift at the Hensen's cells structure the measurement spot indicated contraction after ATP in the zoomed inset (circle and star in the rightmost panel). Scale bar 100 μm. (**K**) Confocal image acquired during an experiment before and after ATP perfusion showed change in morphology identified using Calcein-AM dye (red = before, green = after). Scale bar 100 μm. (**L**) 3D-reconstructions of confocal image stacks displayed an alteration in the morphology after ATP perfusion (green = Calcein-AM, red = Di-3-ANEPPDHQ). Scale bar 50 μm.

In isolated preparations 1-mM ATP was perfused through perilymph. At 82 dB SPL, Hensen's cell vibrations were reduced in most preparations after ATP (Fig. 6A, Inset). On average, the motion amplitude decreased from 128 ± 68 nm to 101 ± 56 nm, measured at the peak (at 193 Hz or 234 Hz) (Fig. 6B; *P* < 0.001, REML one-way ANOVA, mean difference −27 nm, CI −39 to −16, nm, *F*_*2,*__*26*_ = 19, Tukey's multiple comparisons test; n = 21). After ATP washout, partial or full recovery was seen in 70% of preparations (Fig. 6B; *P* = 0.006, REML one-way ANOVA, mean difference −3 nm, CI −22 to −29, Tukey's multiple comparisons test; n = 13). The microphonic potential amplitude decreased as seen from the tuning curves obtained after ATP (Fig. 6C). On average, the CM amplitude decreased from 288 ± 366 μV to 118 ± 197 μV, measured at the peak of each tuning curve (Fig. 6D; *P* < 0.001, mean difference −98 μV, CI −176 to −21, *t* 8, *df* 17, Paired *t*-test; n = 18). ATP caused a minor increase in the electromotility amplitude which decreased to baseline after washout when current steps from −10 to +10 μA were applied (Fig. 6E, Inset). On average, the electromotility amplitude change was non-significant after ATP from 42 ± 29 nm to 52 ± 45 nm (Fig. 6F; *P* = 0.15, RM one-way ANOVA, mean difference 10 nm, CI −6 to27, *F*_*1,*__*12*_ = 2, Tukey's multiple comparisons test; n = 10) and after washout to 48 ± 49 nm (Fig. 6F; *P* = 0.96, RM one-way ANOVA, mean difference −5 nm, CI −31 to21,Tukey's multiple comparisons test; n = 10). ATP-dependent modulation of OHC electromotility may underlie the observed effects on OHC (2, 6) and could be one of several plausible pathways towards protection. Preparations with current steps application suppressed the ATP-induced reduction in motion. Experiments with this current behavioural effect were excluded in the overall motion data statistics in Fig. 6B. The variability likely reflects intrinsic OHCs differences, variation in electrode placement and the sensitivity of laser interferometry to small mechanical and alignment fluctuations.

Non-significant change in the amplitudes of motion from (median 8 nm, iqr 6) to (median 8 nm, iqr 6) was seen at low sound level of 62 dB SPL (Fig. 6G; *P* = 0.85, median difference −0.1 nm, CI −1 to0.3, W 15, Wilcoxon signed-rank test; n = 25). Injection of 5-mM solution of ATP into scala media showed no significant change from 12 ± 8 nm to 13 ± 5 nm in the motion amplitude (Fig. 6H; *P* = 0.47, mean difference 0.2 nm, CI −0.4–0.8, *t* 1, *df* 10, Paired *t*-test; n = 11) serving as a control. No change in the CM amplitude was observed at a moderate sound level or after ATP injection (Supplemental Fig. S3A, B). The EP significantly reduced from (median 5 mV, iqr 8) to (median 1 mV, iqr 3) (Supplemental Fig. S3C; *P* < 0.001, median difference −4 mV, CI −8 to −1, W −223, Wilcoxon signed-rank test; n = 21).

Sound-evoked responses were recorded from a single site within the apical turn (Fig. 6I). A microelectrode was placed inside the scala media near the Hensen's cells to record sound-evoked potentials in a similar way as in Fig. 2. In addition, sound and current-evoked displacements were recorded at Hensen's cells level (red star in Fig. 6I).

Morphological changes after ATP introduction included contraction likely involving Dieter cells level under fluorescence and reflection imaging (Fig. 6J–L). Higher magnification showed contraction (Fig. 6J, right corner panel) across 50% of the preparations.

In summary, we substantiated earlier observations of altered cochlear mechanics and structural changes. The results demonstrated that MET-channel function inside the hearing organ was affected by ATP at high sound levels and this ATP-dependent modulation contributes to the cochlear protective response, a finding that was independently validated using interferometry.

### Perilymphatic ATP decreased calcium levels

**Fig. 7:**
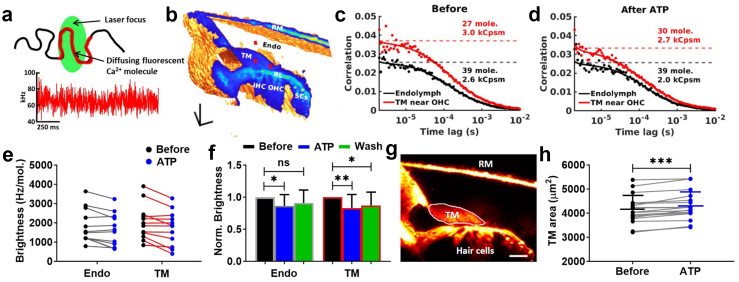
**ATP decreases tectorial membrane calcium determined by FCS.** (**A**) Principle of fluorescence correlation spectroscopy. Ca^2+^ indicator molecules that enter the laser beam focus will be excited and emit red light. As molecules randomly enter and leave the detection volume, the photon count at the detector rapidly fluctuates, as shown in the graph (the *y* axis shows the count rate in kilohertz). (**B**) Measurement positions used in this study: 1. Endolymph i.e. scala media (Endo), tectorial membrane centre (TM), and tectorial membrane near outer hair cells (TM OHC). Scale bar 50 μm. (**C**) Autocorrelation curves obtained from tectorial membrane (TM OHC) (red trace) and endolymph (black trace) before ATP perfusion in an example preparation. Each trace is averaged from 10 recording repetitions, and the fitted model (Equation 1) is illustrated by a continuous line. According to the fits, the number of dye molecules in the detection volume was 39 in endolymph, and 27 in tectorial membrane. Combined with the photon count information of the microscope detector, the molecular brightness values for the Ca^2+^ indicator dye were 2.6 and 3.0 kCpsm, respectively, indicating elevated Ca^2+^ level in TM compared to endolymph. kCpsm, kilocounts per molecule per second. (**D**) Example autocorrelation curves after 1 mM ATP perfusion from the same experiment as in (C), demonstrated a decrease in molecular brightness, and thus, a decrease in Ca^2+^ level, both in TM and in endolymph. (**E**) The effect of ATP perfusion on molecular brightness of the Ca^2+^ indicator in endolymph and TM. Dots represented individual experiments and the recordings from the same experiment are connected by line. For TM, the centre and OHC locations are shown as average recordings. (**F**) Mean molecular brightness from (E) normalised to the corresponding averaged value before ATP (n = 12). Both in endolymph and in TM, Ca^2+^ level was significantly reduced after ATP. The effect showed partial recovery in both locations after washout (n = 11). The two TM locations demonstrated similar behaviour and therefore, they were both included in calculation of general average in TM. (**G**) To quantify the morphological change in TM, an outline was drawn across the TM area (white line). Scale bar 50 μm. (**H**) The area of the tectorial membrane (TM) increased slightly after ATP (n = 17). Data are the means ± SD. ∗∗∗*P* < 0.001; ∗∗*P* < 0.01; ∗*P* < 0.05; n.s., not significant; RM one-way ANOVA followed with post-hoc test and Paired *t*-test applied as appropriate.

To measure free Calcium levels inside the cochlea, a separate set of temporal bones were isolated. A total of 19 animals were used for fluorescence correlation spectroscopy (FCS) experiments: 2 out of 19 preparations served as control (without ATP), 3 were excluded due to damage during microdissection or measurement and 2 datasets were discarded due to weak autocorrelation. A microelectrode containing fluorescent calcium dye Calbryte 590 was placed close to the hearing organ at the apex of the cochlea. After injecting the indicator into endolymph, the calcium-dependent molecular brightness of the dye was obtained by computing the autocorrelation of the fluorescence fluctuations. Calcium levels were measured at two positions in the tectorial membrane, in the centre and in the edge close to the outer hair cells, and a single position in the endolymph (Fig. 7B).^16^ Based on the example correlation curves shown in Fig. 7C and D, 3000 photons were detected per dye molecule per second within the tectorial membrane. The corresponding molecular brightness value for the endolymph was 2600 counts/molecule/s, demonstrating higher calcium in the tectorial membrane than in the endolymph, as shown before (15),.^23^ After ATP introduction, in the same preparation (Fig. 7D), molecular brightnesses in the tectorial membrane and endolymph were reduced, demonstrating calcium level decrease in both. Fig. 7E summarises molecular brightness values from all preparations recorded before and after ATP in tectorial membrane (both the centre and near OHC) and endolymph. The averaged calcium indicator brightness decreased 17% in the tectorial membrane and 14% in the endolymph. The change in brightness was statistically significant for both the TM (centre and OHC combined) (*P* = 0.002) and the endolymph (*P* = 0.049) (Fig. 7F; RM one-way ANOVA, mean difference −216 photons, CI −379 to −54, *F*_*2,*__*21*_ = 3, Tukey's multiple comparisons test; n = 12). After ATP washout, the molecular brightnesses recovered partially, with a trend towards normalisation of Ca^2+^ in the endolymph (*P* = 0.30), but TM calcium remained reduced (*P* = 0.01, RM one-way ANOVA, mean difference −296 photons, CI −495 to −96, *F*_*2,*__*45*_ = 8, Tukey's multiple comparisons) in TM (Fig. 7F) indicating a sustained effect in TM.

Thus, perilymphatic ATP reduced tectorial-membrane calcium levels, a change that may contribute to a Ca^2+^-dependent protective response.

In the experiments, where ATP was perfused into the perilymph, the tectorial membrane displayed minor swelling from 4150 ± 584 μm^2^ to 4298 ± 583 μm^2^ (Fig. 7G and H; *P* < 0.001, mean difference 107 μm^2^, CI 48–165, *t* 5, *df* 16, Paired *t*-test; n = 15). The CM recorded concurrently with Ca^2+^ level determinations at 94 dB SPL reduced from 1022 ± 742 μV to 715 ± 680 μV (n = 13) as observed earlier.

### ATP effects on RM epithelial cells

To study the effect of perilymphatic ATP, we performed whole-cell patch clamp recordings on the cells in Reissner's membrane. A total of 49 animals were used for ATP patch experiments: 10 of 49 preparations were used for training purpose and 23 were considered for statistics. The remaining 16 were discarded due to unhealthy or damaged preparations or no recordings made.

**Fig. 8:**
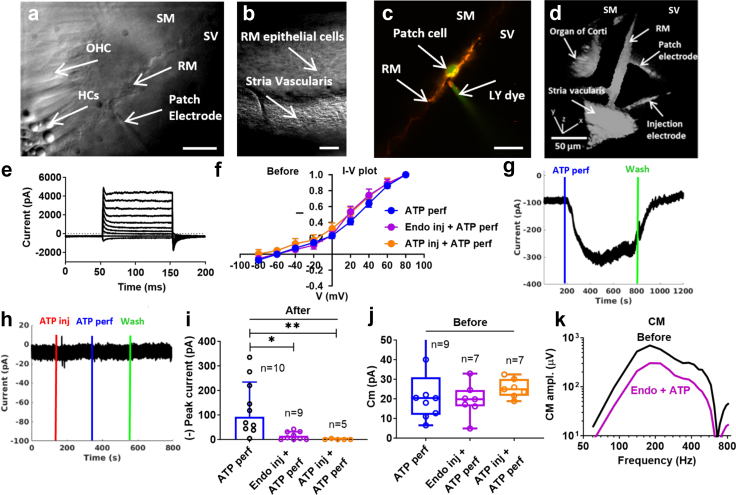
**ATP-gated currents in Reissner's membrane epithelial cells determined by whole-cell patch clamp.** (**A**) Patch camera image showing whole-cell voltage clamp recording from an RM epithelial cell in situ. Scale bar 100 μm. (**B**) Patch camera image with top-down view showing RM epithelial cells attached to stria vascularis. Scale bar 100 μm. (**C**) Confocal image showed the recorded cell stained using Lucifer yellow dye and RM cell boundary stained using di-3-aneppdhq. Scale bar 20 μm. (**D**) 3D-reconstruction of a confocal image under reflection mode showed the patch electrode and ATP injection electrode 200 μm apart in Z-axis focal depth. (**E**) Voltage activated currents before ATP application in an RM epithelial cell (step protocol, −80 to +100 mV, 20 mV increments, 2 s intervals, holding potential −60 mV). Note the outwardly rectifying current. (**F**) Averaged current–voltage relationship derived from (E) demonstrated that injection and/or perfusion of ATP did not affect the voltage-gated currents (n = 5, n = 5, n = 4). The current reverses at −60 to −80 mV, consistent with the activation of a potassium conductance. (**G**) 1 mM ATP perfusion into the scala vestibuli evoked a inward current in RM epithelial cells. (**H**) 5 mM ATP injection into scala media evoked no current in RM epithelial cells, and subsequent perfusion of ATP evoked no detectable current. The vertical lines across the data indicate the time of ATP applications and washout. (**I**) ATP-evoked currents averaged from (G, H) where ATP perfusion after ATP injection did not evoke a detectable current. Data is the median ± IQR. (**J**) Average membrane capacitance from (G, H) under the same three mentioned conditions as shown in (I) demonstrated no significant change. Data is the means ± SD. ∗∗*P* < 0.01; ∗*P* < 0.05; n.s., not significant; Ordinary and Kruskal-Wallis one-way ANOVA followed with post-hoc test applied. (**K**) Cochlear microphonic potential (CM) tuning curves before and after 1 mM ATP perfusion in an example preparation displaying large reduction in CM amplitude.

In the *ex vivo* preparations, RM epithelial cells facing the perilymphatic compartment were recorded. In this study the preparations had preserved polarisation and ionic milieus. An example recording of a RM epithelial cell shown in a cross-section view (Fig. 8A) and RM attachment to stria vascularis in a top-down view (Fig. 8B) confirmed that epithelial cells and not mesothelial cells were recorded.^25^^,^^30^ Confocal imaging performed simultaneously showed the recorded cell stained with Lucifer yellow dye and RM cell boundary stained with red dye di-3-aneppdhq (Fig. 8C). A 3D-reconstruction of confocal images under reflection mode showed the patch electrode located at the centre of the RM and the injection electrode penetrated through RM towards the stria vascularis attachment (Fig. 8D).

The RM epithelial cells had a membrane capacitance (Cm) of 15–50 pF and membrane resistance (Rm) of 300 MΩ−3 GΩ (n = 8). Applying voltage steps at a holding potential of −60 mV before ATP application induced currents with outward rectification evident positive to ∼ −20 mV (Fig. 8E). The voltage activated currents before and after ATP treatments/washout were double in amplitude while the subtracted currents had the same amplitude as before and did not display the inward rectification as seen with ATP-gated currents. These were only basic and not ATP-evoked membrane currents (Supplemental Fig. S3D, E). The currents show similarities to outward currents previously recorded in OHCs^31^ and the current–voltage relationships were consistent with potassium conductance (Fig. 8F). In single RM epithelial cells, perfusion of 1.0 mM ATP entering through scala vestibuli on the outer side of the RM induced inward currents of ∼−50 to −300 pA recorded in voltage clamp at a holding potential of −60 mV (Fig. 8G; n = 8) in contrast to previous findings,^12^^,^^32^ where ATP evoked currents when introduced on the scala media face of RM. We verified the effect by performing additional experiments. When ATP was introduced on both sides of RM (in scala media via injection of 5 mM and in scala vestibuli through perfusion of 1 mM ATP as described in Methods) there was an absence of ATP-evoked currents (Fig. 8H; n = 5). There was a statistically significant difference in the ATP-evoked peak current amplitudes when comparing the current evoked by ATP perfusion alone with the current evoked by ATP on both sides of RM from (median 93 pA ± iqr 192) to (median 7 pA ± iqr 31) (Fig. 8I; *P* = 0.01, Kruskal-Wallis one-way ANOVA, median difference −118 pA, CI −220 to −44, Dunn's multiple comparisons test). However, there was a decline in the currents evoked when artificial endolymph was injected into scala media followed by ATP perfusion, to ∼ (median 0.1 pA ± 2 iqr) (Fig. 8I; *P* = 0.002, Kruskal-Wallis one-way ANOVA, median difference −118 pA, CI −220 to −68, Dunn's multiple comparisons test; n = 9). The variability may arise from intrinsic RM cells differences, variation in membrane capacitance and access resistance. The reduced ATP-evoked currents may be speculated to be caused by activation of other purinergic receptors due to activation of stretch-activated channels when an extra electrode is penetrated through RM,^24^^,^^25^ causing a reduction in the ATP-evoked currents. There was no change in the cell membrane capacitance and series resistance recorded before ATP treatments (Fig. 8J, Supplemental Fig. S3F). These preparations allowed us to record electrical potentials before and after ATP during the experiment. A similar reduction in the CM amplitude after ATP perfusion (Fig. 8K) was recorded.

In summary, perilymphatic ATP evoked inward currents, contradicting the cation-shunt hypothesis and indicating a more complex signalling mechanism. The inward current was neutralised when ATP was applied to both sides of Reissner's membrane, highlighting the importance of ATP flux direction.

Together with the ATP-dependent reduction in RM cell current and TM Ca^2+^, these findings support a model in which ATP signalling modulates cochlear mechanics through ATP-induced depolarisation Ca^2+^-dependent promoting a protective response.

### Effect of TNP-ATP in combination with ATP on the organ of Corti

To verify the efficacy of extracellular ATP seen in the organ of Corti at a high sound level earlier in (Fig. 2, Fig. 3, Fig. 4, Fig. 5, Fig. 6), an ATP derivative called TNP-ATP was used. TNP-ATP is a fluorescently tagged ATP analogue and has a high binding affinity for ATP receptors.^33^^,^^34^ Over the past two decades, TNP-ATP has been widely recognised as a potent and partially selective P2X receptor antagonist.^35^^,^^36^ TNP-ATP in combination with ATP was tested both by perfusion and by injection (Supplemental Fig. S4, S5). Because of its high binding affinity and weak antagonist property TNP-ATP when used in combination with ATP in perilymph fully inhibited the ATP-induced reduced OHC bundle motion and partially the sturdy electrical and endocochlear potentials thus suppressing the modulated cochlear functionality during loud sound exposure (Supplemental Fig. S4). Endolymphatic TNP-ATP with ATP had no effect on sound-evoked responses (Supplemental Fig. S5).

TNP-ATP stained the tectorial membrane, and that signal disappeared in the TM and staining decreased in cochlear cells upon ATP-receptor activation during ATP perfusion and OS as seen earlier, confirming TNP-ATP's inhibitory action (Supplemental Fig. S6). Together with our Ca^2+^ data, these imaging data linked ATP signalling to TM function as a sink and supported the role for the TM in regulating Ca^2+^ as part of a protective response.

Thus, TNP-ATP acted as a low-potency antagonist rather than a clean, selective blocker blocking ATP-evoked change in stereocilia motion and partially inhibiting the effect on electrical potentials.

## Discussion

The present study provides a comprehensive and mechanistically coherent framework for understanding how extracellular ATP modulates cochlear function in a compartment-specific, directionally selective, and receptor-dependent manner. Our findings challenge the long-standing assumption that ATP acts primarily through a classic cation-shunt mechanism across RM. Instead, the data reveal that ATP exerts distinct physiological effects depending on whether it is present in perilymph or endolymph, with perilymphatic ATP emerging as the dominant regulator of sound-evoked responses. This work reframes ATP signalling in the cochlea as a spatially constrained, adaptive process that supports dynamic modulation of cochlear sensitivity at moderate high sound level.

ATP-gated ion channels, particularly P2X2 receptors, were strongly localised on Reissner's membrane (RM) epithelial cells, showing more intense fluorescence staining than in hair cells or supporting cells (e.g., Deiter cells and Hensen's cells)^27^^,^^37^^,^^38^ consistent with prior mRNA labelling in guinea pig and rat cochleae.^29^^,^^39^, ^40^, ^41^ No P2X2 expression was detected in the stria vascularis, spiral ganglion neurons, or nerve terminals, which was consistent with previous work.^2^^,^^42^ Although P2X2 receptors were previously identified on hair-cell stereocilia^29^^,^^43^, ^44^, ^45^, ^46^ and in the basal cochlea,^47^ these locations were not evident in our preparations. Acoustic overstimulation (104 dB SPL) induced structural changes in the organ of Corti (Fig. 1A) and enhanced P2X staining in RM cells (Fig. 1B), consistent with the noise induced increases in P2X2 mRNA.^2^ When perilymphatic ATP was combined with overstimulation (Fig. 1D) fluorescence in hair and supporting cells increased further, indicating ATP-mediated modulation of cochlear micromechanics. Together, these results highlight organ of Corti cells as a major ATP-responsive site supporting the role of extracellular ATP in driving structural and functional changes during acoustic stress.

A central and robust finding is that ATP introduced into the perilymph modulates cochlear mechanics and electrical potentials, towards moderate to high sound levels. ATP significantly influenced cochlear microphonic (CM) and summating potentials (SP) at moderate to high sound levels (70–85 dB SPL; Fig. 2, Supplemental Fig. S1). This SPL-dependence is physiologically meaningful as it suggests that ATP release during loud sound acts as a protective adaptation,^9^^,^^32^^,^^48^, ^49^, ^50^ not a pathological trigger.

The reduction in electrical potentials at 70–85 dB SPL, aligns with the expected time course of ATP accumulation in perilymph and the limited activity of ecto-ATPases.^51^ This temporal match supports a model in which ATP acts as a rapid, reversible modulator of OHC transduction currents and electromotility.^2^^,^^6^ The overshooting recovery of CM and outer hair stereocilia motion after ATP washout indicates that ATP directly shifts the OHC operating point rather than inducing damage.

At high sound levels, perilymphatic ATP altered electrical potentials (Figs. 2 and 6) and OHC stereocilia motion (Fig. 3), and Hensen's cell displacement (Fig. 6) producing measurable changes in organ of Corti mechanics and no change in HC's motion at 65 dB SPL. These effects suggest that ATP reduces the driving force for transduction and shifts OHC input–output functions, likely by rapidly shifting electromotility slightly (Fig. 6) through ATP-induced depolarisation.^52^^,^^53^ ATP-dependent modulation of OHC^2^^,^^6^ could possibly be mediated by Ca^2+^-dependent cytoskeletal changes^54^ or effects on prestin.^55^^,^^56^ Supporting cells also showed ATP-driven structural changes, whereas endolymphatic ATP had no functional impact.^52^^,^^57^ Overall, the data suggest that ATP helps modulate cochlear responses at high sound levels through the underlying mechanisms observed.

Perilymphatic ATP reduced the EP (Fig. 2, Supplemental Fig. S3), partially blocked by TNP-ATP (Supplemental Fig. S4) and consistent with high P2X2 density on Reissner's membrane^2^ despite earlier conflicting reports.^58^ The EP drop likely lowers the MET driving force,^59^ and the results suggest that ATP fine-tunes cochlear responses to protect through converging mechanisms. Because EP was measured only at the beginning and end of experiments, changes may not be fully separated from preparation decline.

Together, these results indicate that perilymphatic ATP acts as a physiologically relevant gain-control signal, modulating cochlear sensitivity and probably OHC function in a manner that is both intensity-dependent and reversible.

In striking contrast, ATP introduced into the endolymph produced minimal or no effects on electrical and endocochlear potentials and stereocilia motion across all sound levels tested (Figs. 4 and 5 and Supplemental Fig. S2). The transient effect may likely result from volume-induced basilar membrane shifts rather than ATP receptor activation.

These findings demonstrate that ATP P2X2 receptors in RM and OHCs are functionally oriented toward the perilymphatic side,^60^ and that endolymphatic ATP despite being present physiologically does not participate in rapid sound-evoked modulation.^61^ Strong expression of P2X2 receptors near the bases of supporting cells such as Dieter cells^48^^,^^62^ and relatively weak expression elsewhere in the cell could contribute to ATP effect in the perilymph and not in endolymph. This asymmetry contradicts the classic cation-shunt hypothesis, which posits that ATP increases conductance across RM into the endolymph.

Although ATP can depolarise hair cells in vitro,^44^^,^^63^ our findings indicate that its functional role is minimal in endolymph but substantial in perilymph, where it can influence hair cells, supporting cells, and possibly the stria vascularis.^11^^,^^64^ Perilymphatic ATP during loud sound likely originates from hair cells, supporting cells, or Reissner's membrane, whereas endolymphatic ATP potentially released from marginal cells^6^^,^^7^^,^^17^^,^^65^ or supporting cells through gap junction hemichannels^6^ does not appear to modulate cochlear mechanics. Thus, ATP-mediated signalling operates primarily outside scala media, reinforcing its compartment-specific nature.

The effects of perilymphatic ATP extended beyond OHCs to supporting cells, including Deiters' and Hensen's cells. These changes likely contribute to the suppression of distortion product otoacoustic emissions (DPOAEs),^66^, ^67^, ^68^ depress cochlear compound action potentials (CAPs) and CM^14^^,^^64^ and shifts in OHC electromotility.^52^

Emerging evidence for P2X7 expression in type II afferents provides an additional layer of complexity. Type II fibres participate in an efferent-like feedback loop that modulates OHC activity at high sound levels.^69^ This mechanism remains possible but cannot account for our results, as the efferent system is inactive in isolated preparations, supporting the idea of designing CIs that directly activate auditory neurons.^70^

Although extracellular ATP has not been directly linked to Ca^2+^-mediated hearing protection, Ca^2+^ is known to regulate hair-bundle stiffness,^71^ and ATP elevates intracellular Ca^2+^ in isolated OHCs through both extracellular entry and release from internal stores.^10^^,^^72^^,^^73^ In our experiments, perilymphatic ATP reduced Ca^2+^ levels in the endolymph and tectorial membrane (TM), consistent with the TM acting as a Ca^2+^ sink (Fig. 7).^16^^,^^23^ This likely reflects rapid ATP-driven Ca^2+^ leakage from enriched microenvironments such as the TM, stereocilia, or the apical hair-cell surface,^16^^,^^74^, ^75^, ^76^ transiently increasing Ca^2+^ within hair bundles and modulating MET-channel-dependent stiffness.^73^ These Ca^2+^-dependent effects link ATP receptor activation to the suppression of CM and stereocilia motion, forming a brief protective response during acoustic stress. The changes occur only with perilymphatic ATP, underscoring that ATP-driven Ca^2+^ signalling is compartment-specific and incompatible with the classic shunt model. By transiently elevating intracellular Ca^2+^, ATP activates downstream pathways that reduce mechanical load on hair cells. This mechanism provides rapid stabilisation of the transduction apparatus under potentially damaging conditions.

Our *ex vivo* cochlear preparation enabled direct in situ patch-clamp recordings from RM epithelial cells, providing functional evidence for compartment-specific ATP signalling. Depolarising steps activated a prominent outwardly rectifying K^+^ current^77^ (Fig. 8E), which increased during recording and is consistent with modulation by ATP-evoked Ca^2+^ release. Imaging confirmed that recordings were obtained from confluent RM epithelial cells rather than mesothelial cells, in line with earlier work showing that only RM epithelial cells express P2X2 receptors and exhibit ATP-gated conductance.^12^^,^^26^^,^^32^

ATP applied from the perilymphatic side evoked slowly desensitising currents^78^ characteristic of P2X2 receptors (Fig. 8G), reinforcing RM's role as a major ATP-responsive surface. In contrast, ATP introduced into the endolymph failed to activate currents (Fig. 8H and I), and prior endolymphatic injection markedly reduced subsequent perilymphatic ATP responses. This suppression is consistent with activation of stretch-activated channels on the endolymphatic surface,^79^ which are sensitive to pressure and ionic shifts^24^^,^^25^ which likely alters membrane tension or ionic homoeostasis, diminishing ATP-gated activity.

These results contradict the classic cation-shunt hypothesis, which assumes ATP acts by increasing conductance across RM from the endolymphatic side. Instead, ATP-evoked currents arise only when ATP is presented to the perilymphatic compartment, while endolymphatic perturbation suppresses rather than facilitates ATP signalling. This polarity-dependent behaviour demonstrates that RM is a directionally sensitive signalling interface, not a passive conduit, and firmly supports a model of compartment-specific ATP signalling independent of the classic shunt.

The use of TNP-ATP, a fluorescent ATP analogue and P2X receptor antagonist,^10^^,^^44^^,^^45^^,^^80^, ^81^, ^82^ provided additional evidence for P2X2-mediated signalling. TNP-ATP acted as a partial antagonist in situ likely due to degradation by ectonucleotidases its ability to suppress ATP-induced changes in stereocilia motion and cochlear potentials (Supplemental Fig. S4) more effectively than suramin^81^^,^^83^, ^84^, ^85^, ^86^ supports the conclusion that ATP acts primarily through P2X2 receptors on OHCs and RM epithelial cells.^2^^,^^45^

**Fig. 9:**
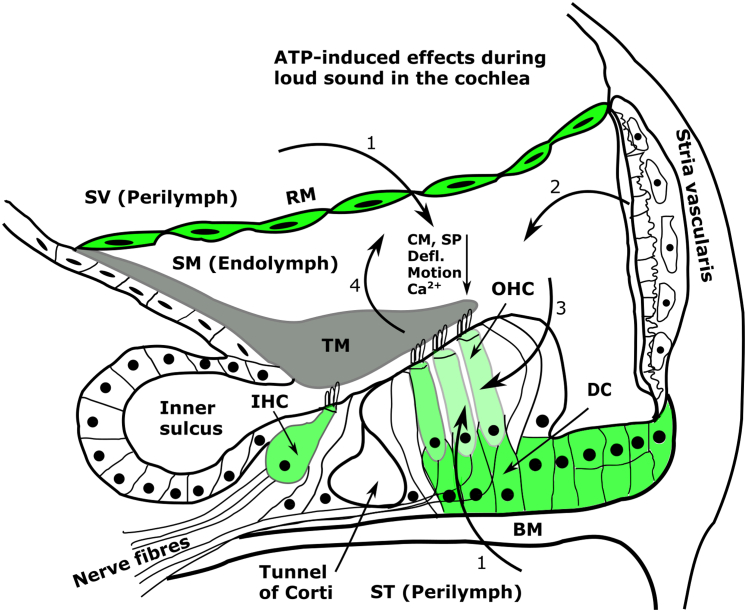
**ATP from the perilymph alters cochlear mechanics and Calcium.** ATP interacts with two distinct binding site populations on Reissner's membrane (RM) epithelial cells via the perilymphatic and endolymphatic sides. Perilymphatic ATP application (1) markedly reduced CM, SP, OHC stereocilia motion, and intracellular calcium, and induced structural changes in the organ of Corti. In contrast, ATP applied to the endolymphatic side (2–4) produced no major functional or structural effect. Yo-Pro-1 uptake increased with perilymphatic ATP, confirming ATP-sensitive receptors in RM epithelial cells, supporting cells (SCs) such as Dieter cells (DCs) and hair cell bodies. Together, these results indicate that perilymphatic ATP activates a protective cochlear response that may help prevent temporary hearing loss during loud sound exposure.

Together, these findings support a model in which perilymphatic ATP acts as the primary upstream signal for cochlear adaptation to moderate loud sound, engaging primarily P2X2 receptors on RM epithelial cells, OHCs, and supporting cells (Fig. 9). This signalling is directional, compartment-specific, and independent of the classic shunt, instead relying on localised receptor activation, Ca^2+^ redistribution,^87^ and modulation of micromechanics. The resulting changes in CM, SP, and stereocilia motion reflect a temporary threshold shift (TTS) that protects the cochlea during moderate loud noise exposure without causing damage.^1^ This signalling provides short-term protection at high sound levels; without it, even brief exposure to otherwise safe low-frequency loud sounds may cause damage and accelerate age-related hearing loss.^23^ The 85-dB exposure used here reflects common human environmental noise^48^ underscoring the relevance of this adaptive mechanism.

Limitations of this study include the use of an *ex vivo* preparation. Previous studies^88^ showed that the passive mechanics of the hearing organ are preserved in these preparations, and cochlear amplification can be restored by current injection.^59^ However, the efferent system that normally controls cochlear function is not functional. Thus, *in vivo* studies are necessary to probe potential efferent effects, but such studies are currently exceedingly difficult. There is also a paucity of human data, so even if ATP effects were demonstrated in mice and guinea pigs, there remains uncertainty as regards its role for human inner ear physiology.

### Conclusions

Our study provides evidence for a regulatory mechanism of extracellular ATP primarily in the perilymphatic compartment modulating cochlear function during loud sound exposure. During loud sound exposure, ATP released into the perilymphatic compartment activates ATP receptors proving direct physiological evidence for ATP's role in the cochlea.

## Contributors

Conceptualisation: S.P., A.F. Supervision: S.P. Study design, project administration, data curation, formal analysis and figures preparation: S.P. Investigation: S.P., U.K., M.P., A.F. Methodology: S.P., U.K., M.P., A.F. Funding acquisition: A.F. Resources and software: S.P., A.F. Literature search: S.P., U.K., M.P. Writing-original draft: S.P. Writing-reviewing and editing: S.P., U.K., M.P., A.F. S.P. performed all the cochlear mechanics experiments, acquired, analysed and interpreted the data. S.P. conducted the patch clamp experiments with the assistance of U.K. and U.K. analysed the data. U.K. have assessed and verified the patch clamp data. S.P. conducted the FCS experiments with the assistance of M.P. and M.P. analysed the data. M.P. have assessed and verified the FCS data. S.P. conducted the laser interferometry experiments and analysed the data with the assistance of A.F. A.F. have assessed and verified the laser interferometry data. All authors read and approved the final version of the manuscript.

## Data sharing statement

All data are available in the main text or the Supplementary Materials. Source data for figures in the article and Supplementary Figures are available on request from the corresponding author.

## Code availability

The computer code for data analysis (Matlab) and acquisition (LabView) are available upon reasonable request.

## Declaration of interests

Authors declare that they have no competing interests.
