# Supplementary material for: Perilymphatic ATP plays a critical role in modulating cochlear function to protect from loud sound induced hearing loss
Source: eBioMedicine. 2026 Jul 14;130:106377. doi: 10.1016/j.ebiom.2026.106377 (PMC13375952; doi:10.1016/j.ebiom.2026.106377)
Supplement: Supplementary Material [file mmc1.pdf]

Supplementary materials for

Perilymphatic ATP plays a critical role in modulating cochlear function to protect from loud sound induced hearing loss

Sonal Prasad*, Urban Karlsson, Marja Pitkänen and Anders Fridberger
Department of Biomedical and Clinical Sciences, Linköping University, SE-581 83 Linköping, Sweden

This PDF file includes:

Supplementary text
Figures S1 to S6

Supplementary text

Fluorescence recovery after photobleaching (FRAP): FRAP was performed using Zeiss FRAP feature integrated in the ZEN 2012 software, by outlining a region of interest on the stained organ of Corti using Yo-Pro-1 dye staining ATP receptors. Photoactivation (uncaging) of caged ATP was performed by illuminating the region of interest with UV illumination at 355 nm which resulted in a rapid and localized release of the free nucleotide at the site of illumination. Following an acquisition of a series of 10 baseline images, a 2-5 µm spot on the hair cell body or Hensen cells was photobleached by focusing the laser at a maximum power into the region of interest. The recovery of fluorescence was tracked by acquiring a series of 30 images at 1- or 3-s intervals over a time of 100–140 s. The images were 512 × 512 pixels, 12-bit pixel depth, with an integration time of 6.30 µs per pixel, and a pinhole of 1.50 Airy units. Confocal images were obtained before and 10 minutes after the over stimulation and caged ATP activation. The experimental data was fitted to a one-phase decay model.

We applied FRAP on the Hensen's cell (HCs) region of interest to determine the ATP uptake by using caged-ATP. As shown Hensen's cells expressed ATP receptor the second highest after RM epithelial cells. Hence, we chose HCs at IHC and OHC region of interest to perform caged-ATP activation by applying FRAP. Identity of receptors was confirmed by using Yo-Pro-1 in parallel with caged-ATP introduction. Upon caged-ATP activation via UV 405 nm laser an increase in the uptake of ATP was observed through increase in the dye intensity in the defined ROI (Fig. S1A; n = 3). The data indicated increased uptake of ATP by its receptors in Hensen's cell at IHC and OHC bodies demonstrating release of ATP.

Perilymphatic ATP effect on sound-evoked responses at 65 dB SPL

The CM amplitude decreased non significantly from 558 ± 241 µV to 401 ± 246 µV (Fig. S1B, C; $P < 0.001$, REML one-way ANOVA, Tukey's multiple comparisons test; n = 12) and recovered significantly to 663 ± 341 µV (Fig. S1C; $P = 0.003$, REML one-way ANOVA, Tukey's multiple comparisons test) above the baseline after washout. In half of the preparations, a shift in the peak of tuning curve towards a higher frequency was observed, which was not noticeable at higher stimulus level. However, the decline in the SP amplitude with flipped polarity (Fig. S1D) after ATP introduction was profound in comparison to the CM change. The SP amplitude decreased significantly from (median 0.8, iqr 0.5) to (median 0.1, iqr 0.2) (Fig. S1E; $P = 0.003$, Kruskal-Wallis one-way ANOVA, Dunn's multiple comparisons test; n =13) with continued small decrease to (median 0.02, iqr 0.1) (Fig. S1E; $P = 0.51$, Kruskal-Wallis one-way ANOVA, Dunn's multiple comparisons test) after washout. There was no alteration in response timing after ATP introduction and washout (Fig. S1F; n = 13), in comparison to before (Fig. S1G). Across 7 preparations, changes in the amplitudes of the tip motion from 85 ± 34 nm to 60 ± 24 nm (Fig. S1H; $P = 0.004$, REML one-way ANOVA, Tukey's multiple comparisons test, after ATP introduction), and to 66 ± 28 nm (Fig. S1H; $P = 0.94$, REML one-way ANOVA, Tukey's multiple comparisons test, after ATP washout) were seen. Base motion amplitude decreased significantly from 109 ± 13 nm to 70 ± 18 nm (Fig. S1I; $P < 0.001$, REML one-way ANOVA, Tukey's multiple comparisons test) but the recovery was non-significant to 89 ± 43 nm) (Fig. S1I; $P = 0.22$, REML one-way ANOVA, Tukey's multiple comparisons test) after washout. Overall, the stereocilia deflection showed a non-significant decrease from 92 ± 33 nm to 72 ± 20 nm (Fig. S1J; $P = 0.25$, REML one-way ANOVA, Tukey's multiple comparisons test) and non-significant increase 89 ± 41 nm (Fig. S1J; $P = 0.68$, REML one-way ANOVA, Tukey's

multiple comparisons test) after washout. No significant change in the electromotility amplitude was seen from 131 ± 34 nm to 169 ± 70 nm (Fig. S1K; $P = 0.63$, RM one-way ANOVA, Tukey's multiple comparisons test) after ATP introduction and to 99 ± 49 nm (Fig. S1K; $P = 0.02$, RM one-way ANOVA, Tukey's multiple comparisons test; n = 5) after washout.

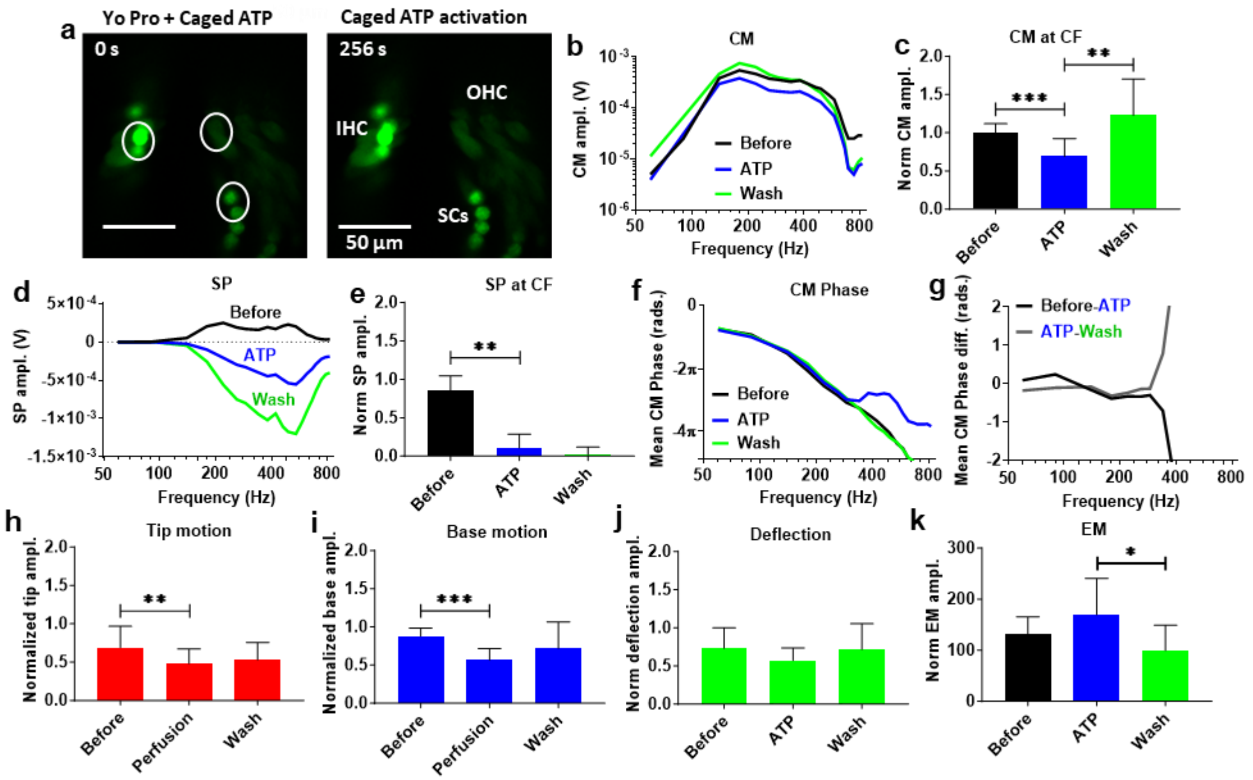

Fig. S1. ATP effect on sound-evoked responses at 65 dB SPL in perilymph. (**A**) Hensen's cells at IHC body and OHC body showed increase in the Yo-Pro-1 dye intensity (within ROI) after caged ATP activation within 4-5 minutes using UV light during FRAP experiment (n = 3). (**B**) Tuning curves of CM before and after 1 mM ATP perfusion, wash in an example preparation. (**C**) Bar graph displaying average CM amplitude response at CF from experiments in (B) showed non-significant decrease after ATP perfusion and bigger significant recovery compared to baseline after washout (n = 12). Data is the means ± SD. (**D**) SP tuning curves before and after ATP perfusion, wash in an example preparation (different experiment than B). Note the large decrease and flip of polarity after ATP perfusion and continued decrease in amplitude after washout. (**E**) Bar graph displaying average SP amplitude response at CF from experiments in (D) showed significant decrease after ATP perfusion and washout (n = 13). Data is the median ± IQR. (**F**) Phases of the CM measured from experiments in (C) relative to the voltage driving the loudspeaker. Note the change phase after ATP perfusion near 400 Hz. (**G**) Mean phase difference between before and after ATP and washout, excluding preparations where the cochlear microphonic potential was close to the noise floor of the recording. (**H**) Bar graphs displaying averaged motion of the outer hair cell stereocilia bundle tip (red bar) showed no change after ATP perfusion. (**I**) Bar graphs displaying averaged motion of the outer hair cell stereocilia bundle base (blue bar) showed significant decrease after ATP perfusion. (**J**) Bar graph displaying averaged outer hair cell stereocilia deflection (green bar) showed no change after ATP perfusion. Data were normalized to the base trajectory amplitude recorded before the ATP perfusion. Averaged data from 7 individual preparations. (**K**) Bar graph displaying averaged electromotility amplitude showed non-significant change after ATP perfusion from 5 individual preparations. All data sets were normalized to the data recorded before perfusion. Data are the means ± SD. ***$P < 0.001$; **$P < 0.01$; *$P < 0.05$; n.s., not significant; RM, REML and Kruskal-Wallis one-way ANOVA followed with post-hoc tests applied as appropriate. The acoustic stimulus was a 200-Hz tone burst at 65-70 dB SPL with ±10 µA.

Endolymphatic ATP had no effect on sound-evoked responses at 65 dB SPL

The CM amplitude measured at the peak of each tuning curve (Fig. S2B) remained unchanged from (median 0.8, iqr 0.3) to (median 0.8, iqr 0.4) (Fig. S2C; $P = 0.80$, Wilcoxon signed-rank test; n = 15) after ATP injection. No significant change in the SP amplitude was observed either (Fig. S2D), decreased on average from (median 0.8, iqr 0.4) to (median 0.5, iqr 0.8) (Fig. S2E; $P = 0.12$, Wilcoxon signed-rank test; n = 15). There was no significant alteration in response timing after ATP (Fig. S2F; n = 15), showing a slight phase lead after ATP in comparison to before (Fig. S2G). No significant changes in the amplitudes of the base motion (from 129 ± 26 nm to 146 ± 59 nm; $P = 0.20$, paired *t*-test), tip motion (from 113 ± 33 nm to 124 ± 48 nm; $P = 0.26$, paired *t*-test), and deflection amplitude (from 99 ± 40 nm to 108 ± 51 nm; $P = 0.40$, paired *t*-test) were seen (Fig. S2H-J, n = 12). Furthermore, there was no significant change in the electromotility amplitude (from 113 ± 46 nm to 93 ± 39 nm; Fig. S2K; $P = 0.14$, paired *t*-test; n = 11).

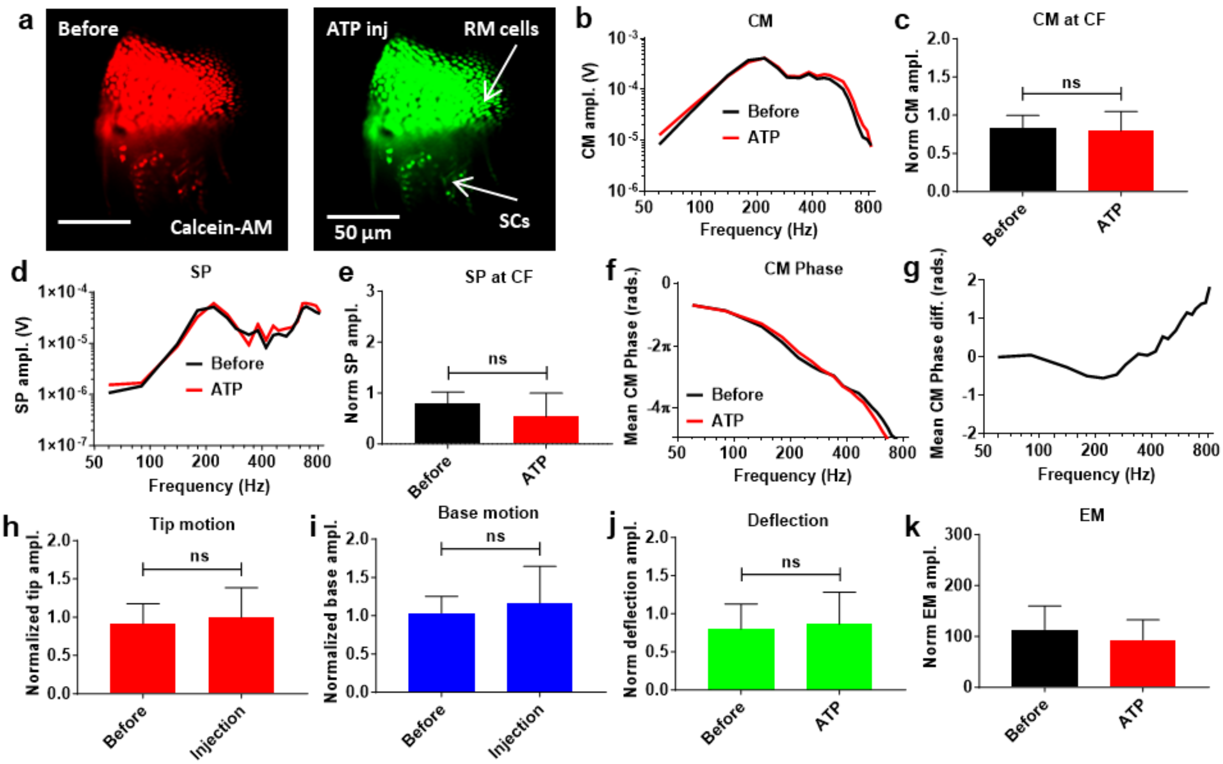

Fig. S2. No ATP effect on sound-evoked responses at 65 dB SPL in endolymph. (**A**) Confocal image acquired during an experiment before and after ATP identified using cytosolic Calcein-AM dye (red = before, green = after). (**B**) Tuning curves for the CM before and after 5 mM ATP injection in an example preparation. (**C**) Bar graph displaying average CM amplitude response at CF from experiments in (B) showed no significant change after ATP injection (n = 15). (**D**) SP tuning curves before and after ATP injection in an example preparation (different experiment than B). (**E**) Bar graph displaying average SP amplitude response at CF from experiments in (C) showed no significant change after ATP injection (n = 15). Data are the medians ± IQR. (**F**) Phases of the CM measured from experiments in (C). Note the slight phase lead after ATP injection. (**G**) Mean phase difference between before and after ATP injection. (**H-J**) Bar graph displaying averaged stereocilia tip (red bar), base (green bar) and deflection (green bar) showed no significant increase after ATP injection. Data were normalized to the base trajectory amplitude recorded before the ATP injection. Averaged data from 12 individual preparations. (**K**) Bar

graph displaying averaged electromotility amplitude showed no change after ATP injection from 12 individual preparations. All data sets were normalized to the data recorded before injection. Data are the means ± SD. n.s., not significant; Wilcoxon signed-rank test and Paired *t*-test applied as appropriate. The acoustic stimulus was a 200-Hz tone burst at 65-70 dB SPL with ±10 µA.

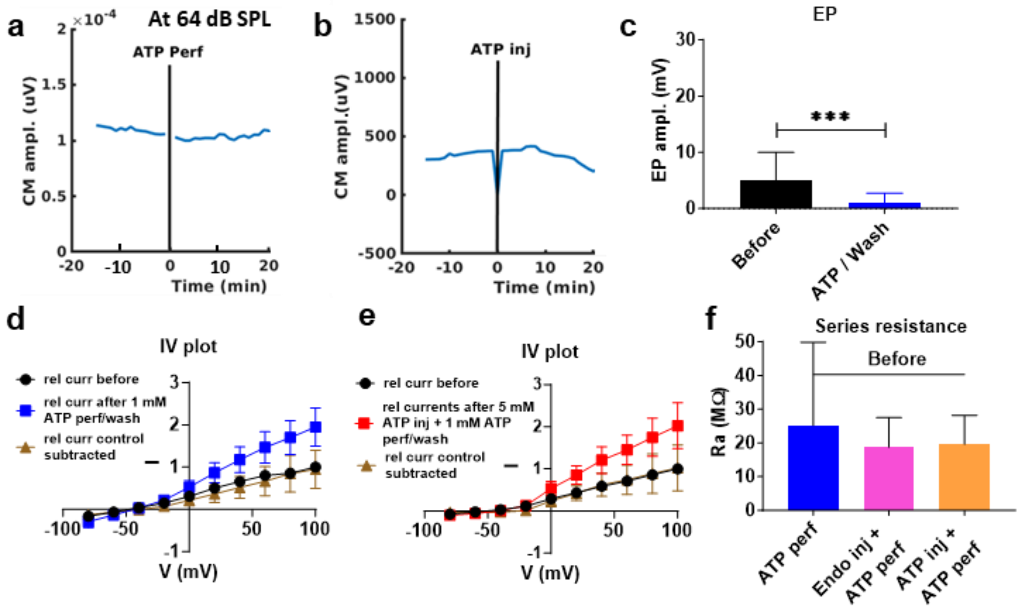

Fig. S3. Laser interferometry electrical potentials recordings and patch-clamp relative currents data after ATP introduction. (**A**) Averaged interpolated data of the CM amplitude (n = 24) remained unchanged after ATP perfusion (black line) at 64 dB SPL. (**B**) Averaged interpolated data of the CM amplitude (n = 10) remained unchanged after ATP injection (black line). (**C**) Bar graph displaying average EP amplitude from experiments in (Fig. 6) showed significant decrease after ATP perfusion / washout (n = 21). Data is the median ± IQR. (**D, E**) Voltage activated K currents during patch experiments demonstrated that before and after ATP treatments/washout (n = 4) doubled in the amplitude, while the subtracted control currents (n = 2) had the same amplitude as before. (**F**) Averages series resistance (Ra) from (Fig. 8G, H) recorded before treatments (n = 8) demonstrated no change under the mentioned three mentioned conditions during patch recordings. Data is the means ± SD. All data sets were normalized to the data recorded before. Wilcoxon signed-rank test and Ordinary one-way ANOVA followed with post-hoc test applied as appropriate.

TNP-ATP blocked ATP-induced suppression of sound-evoked cochlear responses

We investigated the role of TNP-ATP in combination with ATP on sound-evoked electrical responses and outer hair stereocilia motion at a stimulus level of 80 dB SPL near 200 Hz. Except for minor alterations in the brightness of the dye, no morphological changes were observed in stereocilia (Fig. S4A-C). In isolated preparations, the sound-evoked displacement at the tip of the stereocilia (red trajectory) and their base (blue trajectory) showed a minor decrease after TNP-ATP + ATP (Fig. S4A, B). This decrease led to a non-significant reduced deflection amplitude (green trajectory in Fig. S4B). Following the washout, the deflection reversed back to baseline showing complete recovery evident with the larger tip and base motion amplitude (Fig. S4C, note the shapes of the motion trajectories reversed back to normal). Across 7 preparations, there was no significant change in the stereocilia tip motion amplitude (from 96 ± 21 nm to 97 ± 33 nm; $P = 0.99$, REML one-way ANOVA, Tukey's multiple comparisons) after TNP-ATP + ATP perfusion and the washout caused an overshoot to 138 ± 41 nm (Fig. S4D; $P = 0.10$, REML one-way ANOVA, Tukey's multiple comparisons test). There was no significant change in base motion amplitude from 131 ± 14 nm to 112 ± 34 nm ($P = 0.15$, REML one-way ANOVA, Tukey's multiple comparisons test) and an increase to 163 ± 62 nm ($P = 0.06$, REML one-way ANOVA, Tukey's multiple comparisons test) after washout (Fig. S4E). Figure S4F shows no significant change in the deflection amplitude from 84 ± 28 nm to 74 ± 30 nm ($P = 0.34$, REML one-way ANOVA, Tukey's multiple comparisons test) from TNP-ATP + ATP introduction and significant increase to 133 ± 54 nm ($P = 0.04$, REML one-way ANOVA, Tukey's multiple comparisons test) after washout. A large increase in recovery amplitudes after removal of TNP-ATP + ATP points to a similar overshoot phenomenon seen with ATP removal. Current-evoked motility response showed no significant change in the amplitude from 139 ± 64 nm to 109 ± 57 nm (Fig. S4G; $P = 0.60$, REML one-way ANOVA, Tukey's multiple comparisons test; n = 5) after TNP-ATP + ATP introduction and to 122 ± 124 nm ($P = 0.97$, REML one-way ANOVA, Tukey's multiple comparisons) after washout.

The CM amplitude decreased from (median 0.9, iqr 0.8) to (median 0.5, iqr 0.4) (Fig. S4I; $P = 0.008$, Kruskal-Wallis one-way ANOVA, Dunn's multiple comparisons test; n = 7) after TNP-ATP + ATP and increased to (median 0.8, iqr 0.5) (Fig. S4I; $P = 0.23$, Kruskal-Wallis one-way ANOVA, Dunn's multiple comparisons test; n = 4) after washout. The SP amplitude decreased from (median 0.8, iqr 0.3) to (median 0.2, iqr 0.1) (Fig. S4K; $P = 0.008$, Kruskal-Wallis one-way ANOVA, Dunn's multiple comparisons test; n = 7) after TNP-ATP + ATP and the decrease continued to (median 0.1, iqr 0.3) (Fig. S4K; $P > 0.99$, Kruskal-Wallis one-way ANOVA, Dunn's multiple comparisons test; n = 4) after washout. There was a slight alteration in response timing (Fig. S4L; n = 7), showing a slight phase lag after TNP-ATP + ATP and a phase lead after washout towards higher frequencies in comparison to before. A change in the EP amplitude from (median 25, iqr 5) to (median 9, iqr 20), (Fig. S4M; $P = 0.03$, Wilcoxon signed-rank test; n = 8) was seen after TNP-ATP + ATP perfusion.

To conclude, the findings demonstrated that TNP-ATP with ATP suppressed the ATP-induced reduction in outer hair stereocilia motion and partially inhibited the ATP-induced reduction of electrical potentials at a high sound level in comparison to ATP or TNP-ATP alone. This indicated that TNP-ATP partially inhibited the uptake of ATP and its activation.

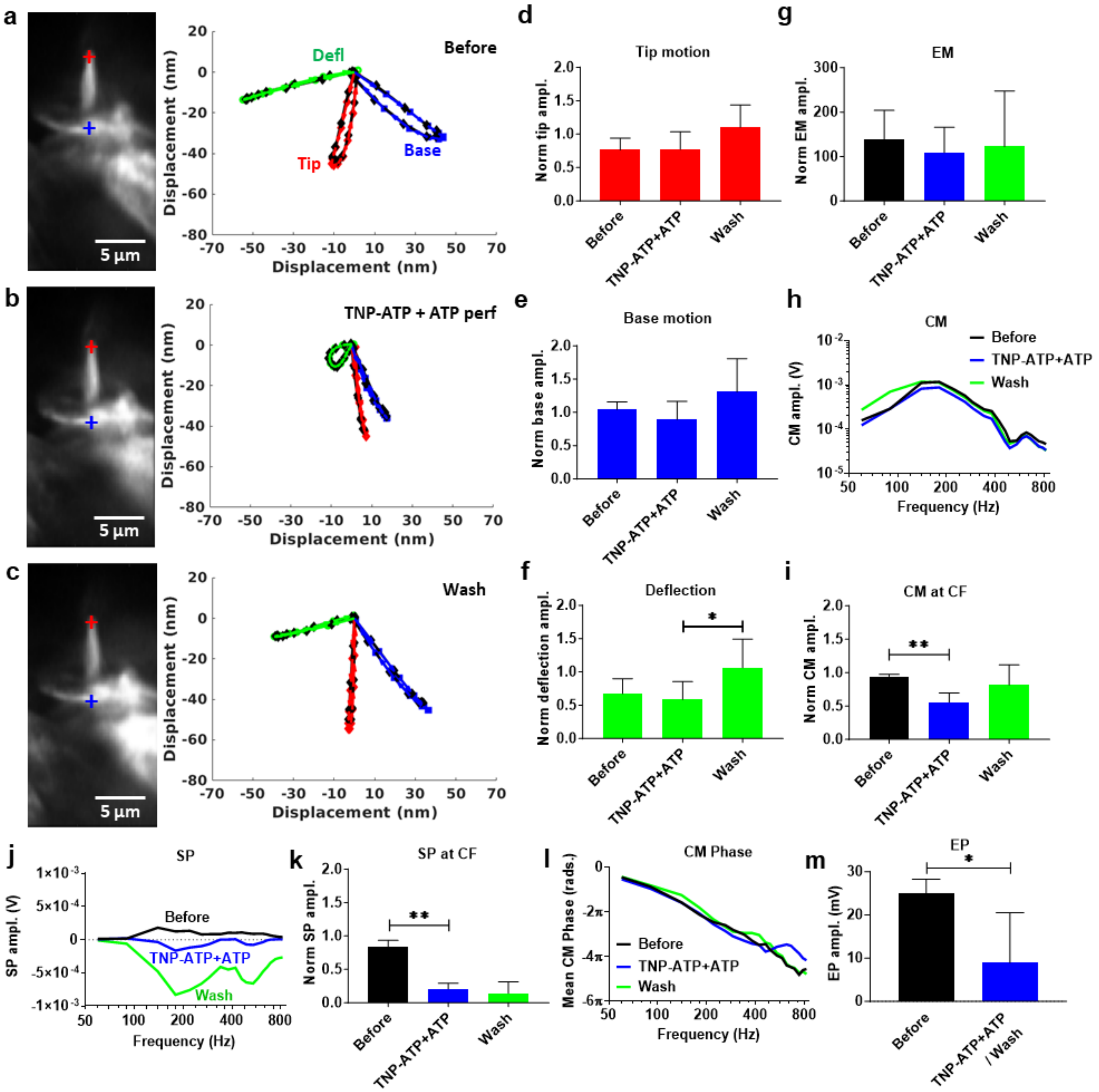

Fig. S4. TNP-ATP + ATP induced inhibitory effect on the reduced sound-evoked responses in perilymph. (**A-C**) Time-resolved confocal image of an OHC stereocilia bundle showing the morphology is intact before and after the 1 mM ATP + 100 µM TNP-ATP perfusion. Representative trajectory data showed non-significant change in sound-evoked motion of the stereocilia tip (red) and base (blue), and deflection (green) after TNP-ATP + ATP perfusion but improvement in the stereocilia motion after washout. (**D-F**) Bar graphs displaying averaged motion of the outer hair cell stereocilia bundle base (blue bar), tip (red bar) and deflection (green bar) after TNP-ATP + ATP perfusion and washout. Data were normalized to the base trajectory amplitude recorded before the TNP-ATP + ATP perfusion. Averaged data from 7 individual preparations. (**G**) Bar graph displaying averaged electromotility amplitude after TNP-ATP + ATP perfusion from 5 individual preparations. The acoustic stimulus was a 200 Hz tone burst at 80 dB SPL with ±10 µA. Data are the means ± SD. (**H**) Tuning curves of CM before and after TNP-ATP + ATP perfusion, and washout in an example preparation. (**I**) Bar graph displaying average CM amplitude response at CF from experiments in (H) showed significant decrease after TNP-ATP + ATP perfusion (n = 7). (**J**) SP tuning curves before and after TNP-ATP + ATP perfusion in an example preparation (same experiment as in H). Note the

amplitude decrease after TNP-ATP + ATP perfusion. (**K**) Bar graph displaying average SP amplitude response at CF from experiments in (J) showed significant decrease after TNP-ATP perfusion (n = 7). (**L**) Phases of the CM measured from experiments in (H) relative to the voltage driving the loudspeaker. Note the change phase after TNP-ATP + ATP perfusion. (**M**) Bar graph displaying average EP amplitude from experiments in (H) showed significant decrease after TNP-ATP + ATP perfusion / washout (n = 8). Data are the medians ± IQR. All data sets were normalized to the data recorded before injection. *$P < 0.05$; n.s., not significant; REML and Kruskal-Wallis one-way ANOVA followed with post-hoc test and Wilcoxon signed-rank test applied as appropriate.

Endolymphatic TNP-ATP with ATP had no effect on sound-evoked responses

TNP-ATP in combination with ATP dissolved in artificial endolymph was injected inside scala media. No morphological changes were observed in stereocilia (except for minor changes in the brightness of the dye, Fig. S5A-B). In an example preparation shown in (Fig. S5A, B), the motion at the tip of the stereocilia (red trajectory) and their base (blue trajectory) remained same leading to no change in the deflection (green trajectory) after TNP-ATP + ATP at a sound stimulus of 80 dB SPL near 200 Hz. No significant change in the base motion from 129 ± 10 nm to 114 ± 56 nm ($P = 0.29$, paired *t*-test), tip motion from 102 ± 14 nm to 98 ± 44 nm ($P = 0.72$, paired *t*-test) and deflection amplitude from 99 ± 40 nm to 108 ± 51 nm (Fig. S5C; $P = 0.17$, paired *t*-test; n = 6) were observed. Electromotility amplitude remained unchanged from 88 ± 24 nm to 68 ± 40 nm (Fig. S5D; $P = 0.15$, paired *t*-test; n = 6). The CM amplitude remained unchanged (Fig. S5E) from 301 ± 252 µV to 192 ± 109 µV (Fig. S5F; $P = 0.08$, paired *t*-test; n = 8). SP amplitude was unaffected (Fig. S5G) and on average there was no significant change from -16 ± 191 µV to -14 ± 109 µV (Fig. S5H; $P = 0.16$, paired *t*-test; n = 8). TNP-ATP + ATP-induced no significant change in the EP from 5 ± 4 mV to 3 ± 4 mV, (Fig. S5J; $P = 0.38$, paired *t*-test; n = 7).

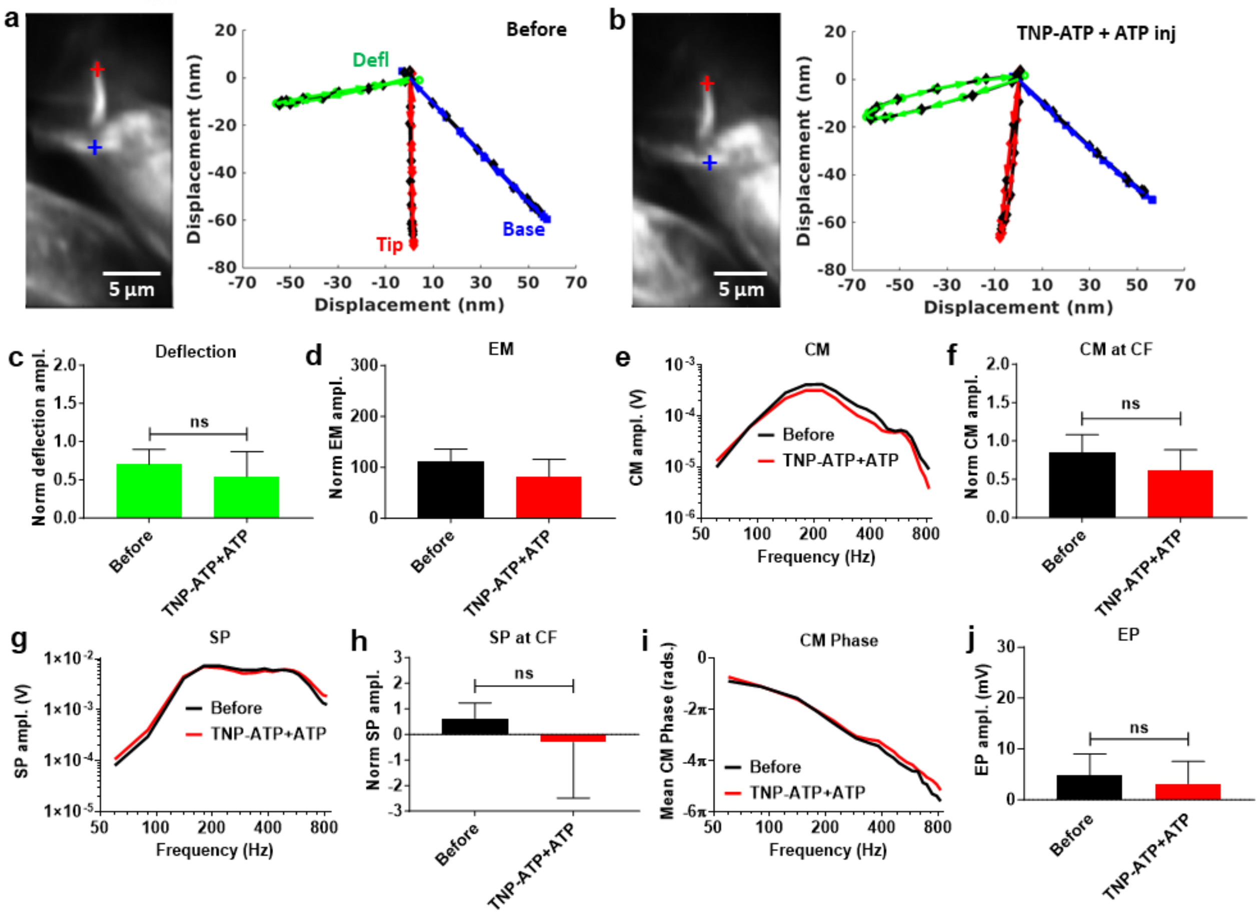

Fig. S5. No TNP-ATP + ATP effect on the sound-evoked responses in endolymph. (**A, B**) Time-resolved confocal image of an OHC stereocilia bundle showing the morphology is intact before and after the 5 mM ATP + 500 µM TNP-ATP injection. Representative trajectory data showed no change in sound-evoked motion of the stereocilia tip (red) and base (blue), and in deflection (green) before and after TNP-ATP + ATP injection. (**C**) Bar graph displaying averaged stereocilia base deflection (green bar) showing no change after injection. Data were normalized to the base trajectory amplitude recorded before the TNP-ATP + ATP injection. Averaged data from 7 individual preparations. (**D**) Bar graph displaying averaged electromotility amplitude after TNP-ATP injection from 6 individual preparations. The acoustic stimulus was a 200 Hz tone burst at 80 dB SPL with ±10 µA. (**E**) Tuning curves of CM before and after TNP-ATP + ATP injection in an example preparation. (**F**) Bar graph displaying average CM amplitude response at CF from experiments in (E) showed no significant change after TNP-ATP + ATP injection (n = 8). (**G**) SP tuning at CF curves before and after TNP-ATP + ATP injection in an example preparation (different experiment than E). (**H**) Bar graph displaying average SP amplitude response at CF from experiments in (G) showed no significant change after TNP-ATP + ATP injection (n = 8). (**I**) Phases of the CM measured from experiments in (E). (**J**) Bar graph displaying average EP amplitude from experiments in (E) showed no significant change after TNP-ATP + ATP injection (n = 7). Data are the means ± SD. All data sets were normalized to the data recorded before injection. n.s., not significant; Paired *t*-test applied as appropriate.

Effect of TNP-ATP in combination with ATP on the organ of Corti

Perfusing TNP-ATP in combination with ATP led to a minor structural change in the supporting cells region (Fig. S6A, B). 50 μM of TNP-ATP alone stained tectorial membrane (Fig. S6C) when perfused. Staining disappeared in TM and reduced in hair cells after TNP-ATP washout (Fig. S6C, second panel). Figure S6D and E showed a decline in CM amplitude after TNP-ATP introduction within 2-3 minutes (n = 2) and recovery after removal.

TNP-ATP with ATP injection led to minor structural changes at hair cells (Fig. S6F, G). 50 μM TNP-ATP with Yo-Pro-1 when injected stained TM and Yo-Pro-1 stained the supporting cells and hair cells (Fig. S6H; n = 2). ATP perfusion combined with overstimulation led the staining disappear in TM (Fig. S6H, second panel) similar as seen after TNP-ATP washout effect (Fig. S6C, second panel) earlier. Staining reduced in hair cells and supporting cells confirming antagonist effect of TNP-ATP contrary to increased staining seen earlier in (Fig. 1D, second panel). CM showed no change within 2-3 minutes of TNP-ATP injection (Fig. S6I, J; n = 2) while the CM dropped drastically after overstimulation (Fig. S6I, J). The results indicated a correlation between ATP and TM as shown with FCS experiments and a possible role of TM in regulating calcium during loud sound exposure described in (Strimbu et al., 2019).

TNP-ATP behaved as a partial antagonist. It blocked ATP-evoked stereocilia motion but did not fully inhibit the electrical potentials and may require higher concentration. These mismatched electrical effects likely reflect ectonucleotidase-mediated degradation, limited tissue access, and parallel ATP actions on supporting and sensory epithelial cells as well as the tectorial membrane. In the intact cochlea, TNP-ATP therefore acted more like a low-potency antagonist with occasional agonist-like behavior rather than a clean, selective blocker.

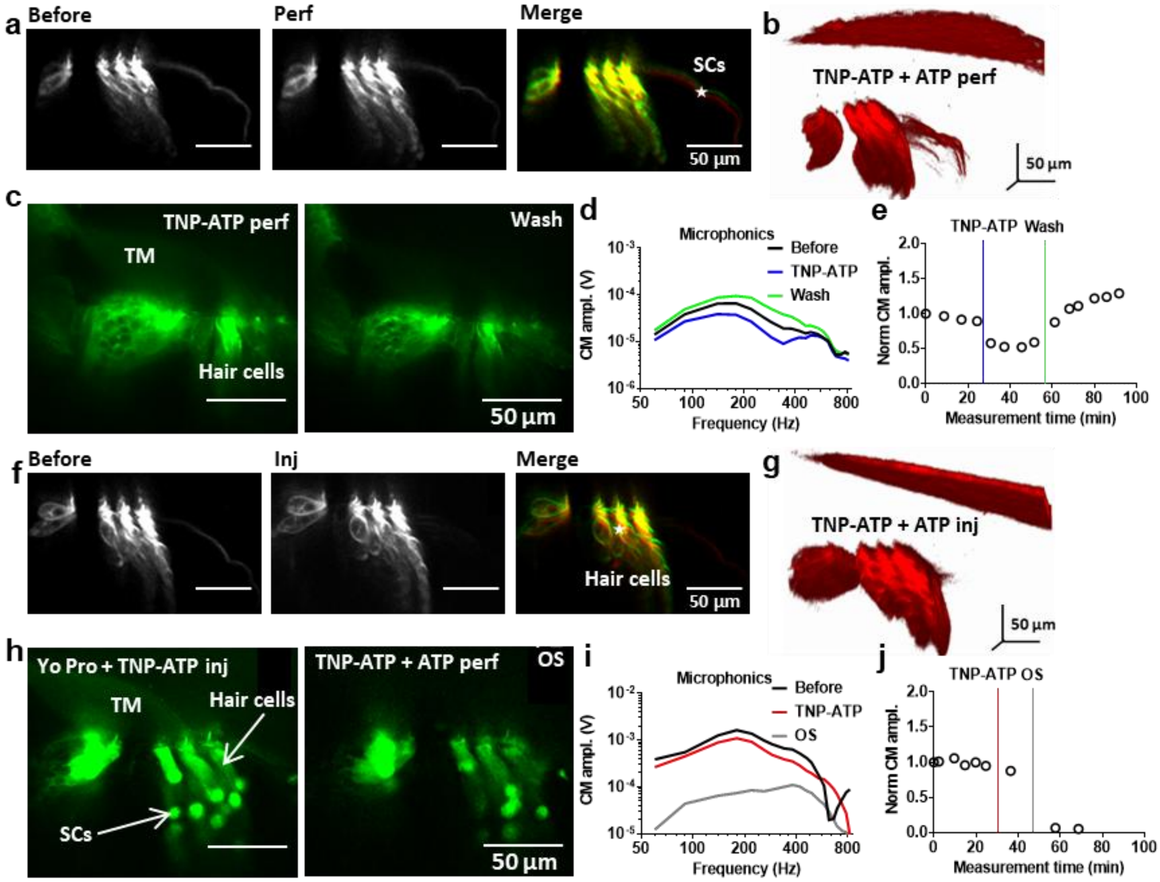

Fig. S6. TNP-ATP + ATP effect on the morphology of the organ of Corti. (**A**) Confocal images showed slight contraction at supporting cells level when TNP-ATP in combination with ATP is perfused. (**B**) A 3D reconstruction of confocal image stacks shown after perfusion. (**C**) When TNP-ATP is perfused tectorial membrane staining was seen, and the staining disappeared after washout (n = 2). (**D**) Tuning curves of CM before, after TNP-ATP perfusion and after washout in an example preparation (n = 2). (**E**) Time course of the normalized CM amplitude showed reduction after TNP-ATP perfusion and large recovery after washout (same preparation in D). The vertical line indicates the time of perfusion and washout. (**F**) Confocal images showed negligible morphological changes at supporting cells level when TNP-ATP in combination with ATP was injected into scala media. (**G**) A 3D reconstruction of confocal image stacks shown after injection. (**H**) Staining of TM seen after TNP-ATP injection, and the staining disappeared, and uptake of Yo-Pro-1 dye was reduced upon ATP perfusion combined with overstimulation. The phenomenon was similar to washout observed in (C). The acoustic stimulus used was a 160-Hz continuous tone burst at 104 dB SPL for 10 minutes for overstimulation (n = 3). (**I**) Tuning curves of CM before and after TNP-ATP injection and after OS in an example preparation. (**J**) Time course of the normalized CM amplitude showed a large reduction after OS (same preparation in I) (n = 2). The vertical line indicates the time of injection of TNP-ATP and OS. The acoustic stimulus used was 80 dB SPL for microphonic potential.
